# Analysis and Application of European Genetic Substructure Using 300 K SNP Information

**DOI:** 10.1371/journal.pgen.0040004

**Published:** 2008-01-18

**Authors:** Chao Tian, Robert M Plenge, Michael Ransom, Annette Lee, Pablo Villoslada, Carlo Selmi, Lars Klareskog, Ann E Pulver, Lihong Qi, Peter K Gregersen, Michael F Seldin

**Affiliations:** 1 Rowe Program in Human Genetics, University of California Davis, Davis, California, United States of America; 2 Department of Biochemistry, University of California Davis, Davis, California, United States of America; 3 Department of Medicine, University of California Davis, Davis, California, United States of America; 4 Broad Institute of Harvard and the Massachusetts Institute of Technology, Cambridge, Massachusetts, United States of America; 5 Division of Rheumatology, Allergy and Immunology, Brigham and Women's Hospital, Boston, Massachusetts, United States of America; 6 The Robert S. Boas Center for Genomics and Human Genetics, Feinstein Institute for Medical Research, North Shore LIJ Health System, Manhasset, New York, United States of America; 7 Center for Applied Medical Research, University of Navarra, Pamplona, Spain; 8 Division of Internal Medicine, San Paolo Hospital School of Medicine, University of Milan, Milan, Italy; 9 Karolinska University Hospital, Stockholm, Sweden; 10 Department of Psychiatry and Behavioral Sciences, Johns Hopkins School of Medicine, Baltimore, Maryland, United States of America; 11 Department of Public Health Sciences, University of California Davis, Davis, California, United States of America; University of Chicago, United States of America

## Abstract

European population genetic substructure was examined in a diverse set of >1,000 individuals of European descent, each genotyped with >300 K SNPs. Both STRUCTURE and principal component analyses (PCA) showed the largest division/principal component (PC) differentiated northern from southern European ancestry. A second PC further separated Italian, Spanish, and Greek individuals from those of Ashkenazi Jewish ancestry as well as distinguishing among northern European populations. In separate analyses of northern European participants other substructure relationships were discerned showing a west to east gradient. Application of this substructure information was critical in examining a real dataset in whole genome association (WGA) analyses for rheumatoid arthritis in European Americans to reduce false positive signals. In addition, two sets of European substructure ancestry informative markers (ESAIMs) were identified that provide substantial substructure information. The results provide further insight into European population genetic substructure and show that this information can be used for improving error rates in association testing of candidate genes and in replication studies of WGA scans.

## Author Summary

Ancestry differences corresponding to ethnic groups may be important in determining disease risk factors and optimizing treatment. Our study further defines ancestry relationship among different European ethnic groups by examining over 300 thousand variations in DNA, in over 2,000 individuals. This study allowed a clearer ascertainment of differences that could not be discerned in smaller studies using more limited numbers of DNA variations. We show clear differences among European American participants of different self-identified ethnic affiliation. The analyses showed multiple components of variation. The components showing the largest variations generally corresponded to the grandparental country or region of origin within Europe. We also show the importance of applying this information in determining genetic risk factors for complex diseases. Moreover, the results have enabled a better selection of smaller numbers of DNA variations that can be used in future disease studies to identify more homogenous participant groups and minimize false positive and false negative results in assessing genetic risk factors for disease.

## Introduction

Differences in population genetic structure and substructure between cases and controls can lead to false positive association tests [1–5]. Interest in this issue has accelerated with the application of whole genome association (WGA) screens for deciphering the genetics of complex diseases. The importance of recognizing and controlling for population structure is magnified when population controls are not closely matched to cases, a process that requires multiple demographic considerations and similar sample acquisition methods. These conditions are difficult and often not practical to fulfill completely. Since many studies focus on participants of European descent, the potential impact of European substructure on association testing has specifically engendered interest [6,7]. In fact, the current study was undertaken as part of an effort to effectively ascertain and adjust for differences in population substructure among cases and controls in our studies of the genetics of rheumatoid arthritis in a participant set that predominantly includes participants of European descent.

Recent studies have addressed differences in population substructure and methods to control for these differences in association testing [8–13]. Population substructure can be explored and ascertained using a variety of algorithms that apply principal component analysis (PCA) or non-hierarchical cluster analysis based on allele frequencies in individuals and groups. Unlike other multi-locus adjustments (e.g. genomic control methods [14]) these newer approaches adjust for the fact that some SNPs have large frequency variations across different populations compared to other SNPs [11]. The ability of these methods to control for large differences in population substructure has been at least partially demonstrated by both real data and simulations [6,11,12]. However, the practical application of these methods and limitations requires more extensive exploration in a variety of real datasets.

Recent studies by our group and others have led to the identification of SNP subsets that can provide European substructure information [6,7]; this is consistent with previous work suggesting distinct clines of genetic variation within Europe [15–20]. These European substructure ancestry informative markers (ESAIMs) may be particularly important in large replication studies in which independent sets of case and control genotypes are necessary to confirm and further define associations without the benefit of genome-wide SNP typing. Previous studies have been limited to initial SNP genotyping sets of less than 10,000 SNPs [6,7]. The current study uses 300K to 500K genome-wide SNP data to enhance the ability to define elements of European substructure that were not evident or poorly defined using smaller sets of SNPs.

## Results

### Principal Component and Cluster Analyses Show Major Differences between European Populations

A set of 952 self-identified participants of diverse European descent genotyped with >300K SNPs was used for the first phase of European population substructure analysis. This participant group predominantly included European Americans as well as smaller numbers of individuals from Italy and Spain (see Methods). In order to reduce potential noise created by continental admixture this study included only those individuals who did not have evidence of non-European continental ancestry (see Methods). The genotypes were examined using the principle component analysis (PCA) algorithm implemented in the EIGENSTRAT program [11], a computational method that enables rapid analyses of very large datasets. Using multiple criteria including ANOVA, a split half reliability test (see Methods) and a test for normality of distribution, substructure was present in multiple principle components (Table 1). However, most of the variance among the populations was observed in the first principal component (PC). This PC accounted for >5 fold the variance of the second PC.

**Table 1:**
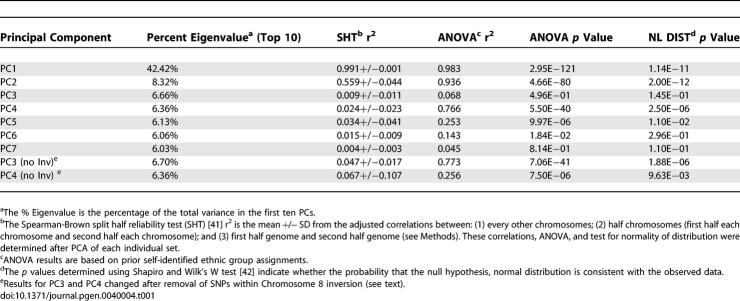
Evaluation of Principal Components Analyses in European Populations Using 300 K SNPs

**Figure 1:**
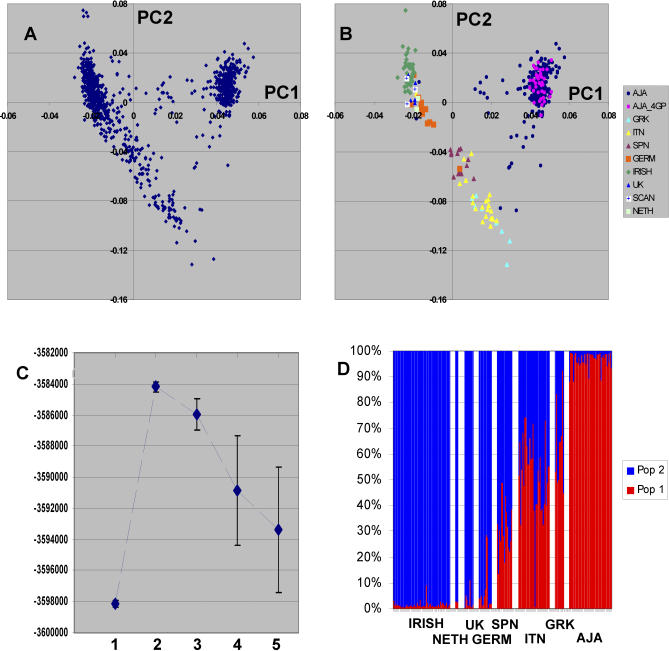
European Substructure Analysis of a Diverse Set of Individuals of European Descent (A) Graphic representation of the first two PCs for 952 individuals genotyped with 300K SNPs. (B) Color code shows subgroup of individuals with more detailed grandparental origin information. Each color-coded individual had 4GP of origin information with the exception of the AJA group. The individuals included 14 Spanish (SPN), 28 Italian (ITN), eight Greek (GRK), 11 German (GERM), 52 IRISH, five United Kingdom (UK), three Scandinavian (SCAN), and two Netherland (NETH). For the Ashkenazi Jewish individuals, 38 had 4GP information (AJA_4GP), and 220 participants were self identified as Ashkenazi Jewish (AJA) but without other information. (C) The STRUCTURE analyses shows results from the same participant set using three random sets of >3,500 SNPs for assessment of the number of population groups (K). The ordinate shows the Ln probability (mean +/− SD) corresponding to the number of clusters. (D) STRUCTURE results under the assumption of two population groups (K = 2). The proportion of each cluster group (population) for each individual is shown by the color code.

The clustering of individuals for PC1 and PC2 corresponded to self-reported regional and ethnic origins (Figure 1A and 1B). This is best illustrated when considering only those participants with the same grandparental country of origin and those individuals that indicated Ashkenazi Jewish ancestry (Figure 1B). Similar to our previous studies using smaller sets of SNPs, the clustering of individuals of Ashkenazi Jewish ancestry does not correspond to grandparental European country of origin, which was diverse [6].

The first PC showed a gradient that distinguished “southern” or Mediterranean origin from “northern” European ancestry (Figure 1B). The mean +/− SD of the first PC scores for those individuals with the same (or adjoining for Scandinavian) 4 grandparent (GP) country of origin or 4GP Ashkenazi ancestry information were: Irish (51 individuals), mean −0.022 +/− 0.002; Scandinavian (3 individuals), mean, −0.022 +/− 0.002; United Kingdom (5 individuals), −0.020 +/− 0.002; German (11 individuals), −0.016 +/− 0.004; Spanish (14 individuals), 0.004 +/− 0.003; Italian (28 individuals), 0.015 +/− 0.006; Greek (9 individuals), 0.022 +/- 0.011; and Ashkenazi (38 individuals), 0.045 +/− 0.003. For participants self-identified as of Ashkenazi heritage, but who lacked 4 GP information (234 individuals), the mean PC1 score value was 0.043 +/− 0.008.

The same dataset was also examined using a Bayesian clustering algorithm (STRUCTURE) [21]. For these analyses we examined three sets of >3500 SNPs that were selected randomly except for the criterion that the minimum inter-SNP distance was >500 Kb (see Methods). This was done to both ensure genome-wide distribution and eliminate linkage disequilibrium between SNPs. This analysis similar to our previously reported studies was most consistent with two population groups (K = 2) explaining the major substructure in this set of European individuals (Figure 1C). The distribution of the individuals (K = 2) was similar to that shown on the first axis of the PCA (Figure 1D) and the individual population contributions were highly correlated with the first PC scores (r^2^ > 0.95 for each of the three random sets compared with the for the 500K SNP data analyzed by PCA).

**Figure 2:**
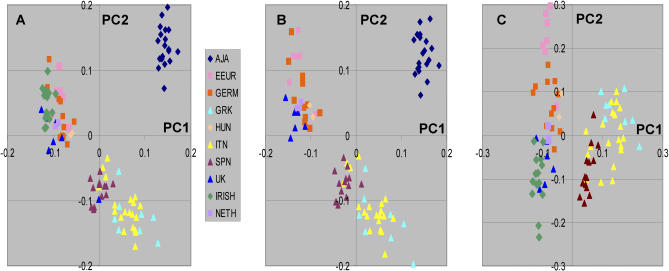
Comparison of Principal Component Analysis Excluding Different Individual Groups Color key shows groups as defined in Figure 1. (A) All individuals with 4GP information. (B) Same individual set except exclusion of individuals of Irish descent. (C) Same individual set with exclusion of Ashkenazi Jewish individuals.

We also explored whether the PCA was affected by either inclusion or exclusion of specific population groups or the number of individuals in different population groups. Most prominently, a major difference in the relationships among the populations for the second PC was observed when either Ashkenazi Jewish individuals or Irish individuals were excluded (Figure 2). These results suggest some caution in interpretation of specific clines and particular relationships among different European groups (see discussion).

### Identification of SNPs Distinguishing Northern from Southern European Origin

For many association tests including candidate genes and replication studies for candidate chromosomal regions it is useful to identify smaller numbers of SNPs that can distinguish European substructure. Previous studies including our own utilized genome-wide SNP sets of ≤10K SNPs. To identify a more robust set of SNPs that could distinguish the largest component of substructure observed in the current data we used the genotypic differences observed in >300K SNPs between two groups of individuals, 150 Ashkenazi Jewish and 125 Northern European individuals. The Ashkenazi Jewish individuals were chosen since 1) this individual group was most clearly distinguishable from the Northern European individuals, 2) might more closely represent an “older” population of Mediterranean origin and 3) we had substantial number of genotyped individuals to enable a good representation of this population. To select the most informative SNPs distinguishing between these groups we determined the informativeness (I_n_) [22] for each of >300K SNPs. The 20,000 SNPs with the highest I_n_ values were then selected to capture the most informative SNPs. To ensure both a more uniform genome-wide distribution and minimize linkage disequilibrium the set of putative European substructure ancestry informative markers (ESAIMS) were chosen to obtain the markers with highest I_n_ with a minimum inter-SNP distance >500 Kb. This resulted in a set of 1441 SNPs (Table S1).

**Figure 3:**
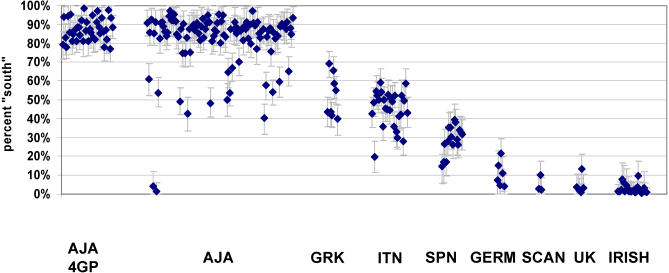
STRUCTURE Analysis Using 1,400 ESAIMs Selected for North/South Information Analysis was performed without any prior population assignment using STRUCTURE under the assumption of two population groups (K = 2). The results are shown for only individuals not used in selection of the north/south-ESIAMs. The individual individuals and 90% confidence limits are shown for selected groups with ethnic and grandparental origins (see Figure S1 for entire results). The individuals grouped by self identification included: Ashkenazi 4GP; Ashkenazi Jewish (without 4GP information) (AJA); Greek (GRK); Italian (ITN); Spanish (SPN); German (GERM); Scandinavian (SCAN); United Kingdom (UK); and Irish.

The STRUCTURE results (K = 2) from individuals with 4 grandparental data (not used for ESAIM selection) showed separation of most of the 220 self-identified individuals of Ashkenazi Jewish heritage (mean 83% south; median, 87%) from 37 individuals of Western, Northern or Central heritage belonging to the “northern” group (mean 4% south; median, 3%), and 51 individuals of Greek, Italian, or Spanish origin were intermediate (mean 41% south; median, 42%) (Figures 3 and S1). These 1441 north/south-ESAIM showed small confidence limits in the assignments; of the total of 677 individual individuals not used in ESAIM selection the maximum 90% Bayesian confidence interval (CI) was 21.1% (e.g. 13.7 % south, 90% CI 2.6 % – 23.0 %) and the median CI was 13.9%. Smaller north/south-ESAIM sets showed strong correlations with the 1441 set e.g. 384 ESAIMs (r^2^ = 0.970) (Figure S2). However, the smaller north/south-ESAIM sets showed somewhat broader confidence limits (e.g. 384 north/south ESAIM set showed maximum CI = 38.9% and a median CI = 17.1%. However, these differences are unlikely to affect most studies. The larger number of north/south-ESAIMs may be useful if a very homogeneous set of individuals of a particular ethnic group is desired for a specific study.

### Further Analysis of Northern European Populations

Although the STRUCTURE analysis was most consistent with two population groups explaining most of the substructure within Europe, the distribution of individuals from different countries of origin along the second axis in the PCA (Table 2; Figure 1B) suggested that further analysis of substructure was warranted. This substructure was examined using individuals of “northern” European ancestry in the context of a large dataset of rheumatoid arthritis cases and controls (over 2000 total individuals) that were recently genotyped with >500K SNPs as part of the NARAC studies (see Methods). For these PCA we examined only those European individuals that showed >90% membership in the northern European group by STRUCTURE analysis using the 1441 north/south-ESAIMs. This criterion closely matched the individual distribution along the first principal component axis of this dataset (Figure S3). Controlling for this first vector in analysis of cases vs. controls decreased the inflation of the median chi-square distribution using the genomic controls parameter (λ_gc_) from 1.43 to 1.15.

**Table 2:**
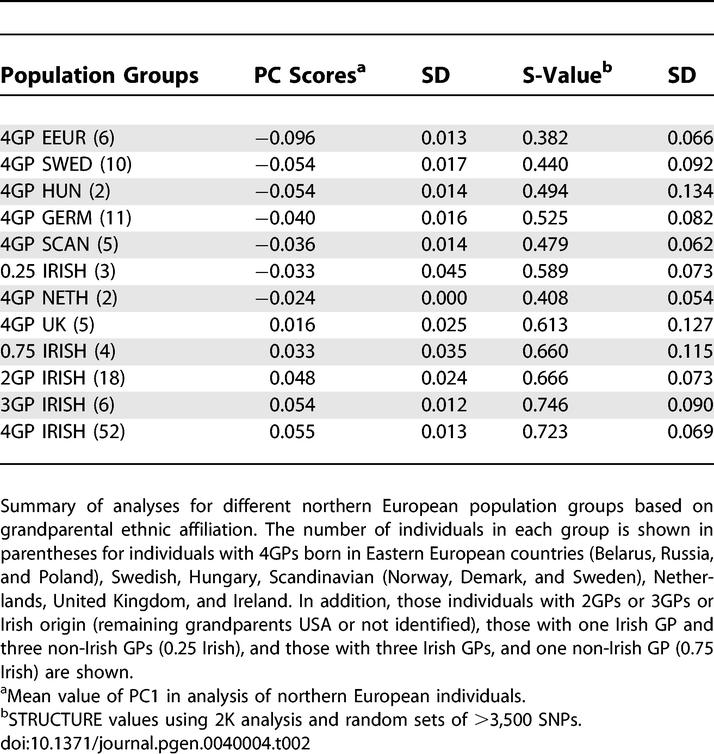
Summary of Principal Component and STRUCTURE Results for Northern European Population Groups

**Figure 4:**
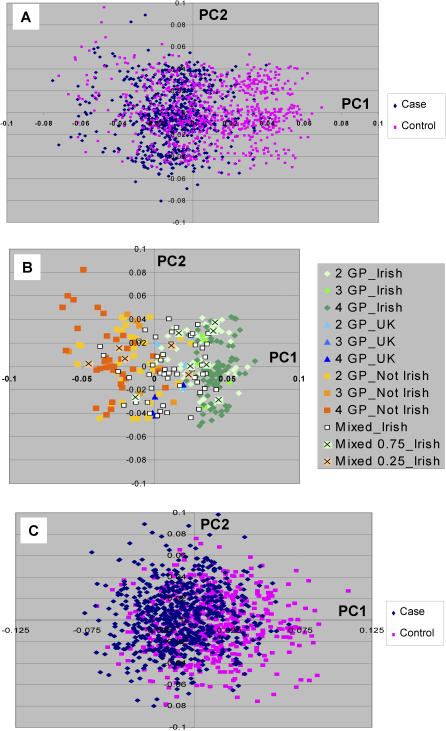
Analysis of European Substructure in Northern European Individuals (A) The first two PCs are depicted for RA cases and NYCP controls. (B) Color codes show the Irish contribution to each individual with at least two GP country of origin information in the sample set shown in (A), e.g., the 2GP Irish individuals have 2GP Irish origin and 2GP unknown or USA origin; Not Irish includes only individuals without known Irish ancestry and with at least 2GP information; mixed Irish are those individuals with at least one GP Irish and one GP non-Irish. (C) Analysis using 1,211 ESAIMs selected for differences along PC1 in northern European individuals (see Results).

PCA of the “north” only subset showed substantial substructure differences in the distribution of North American Rheumatoid Arthritis (NARAC) cases and controls along the first PC (Figure 4). Importantly, we controlled for this difference in our genome-wide association scan and excluded SNPs that showed association based on this substructure difference [23]. The distribution of individuals in this PC showed a distinct pattern with respect to the context of country of origin information that was available for a subset of control individuals (Figure 4B). Most notably, Irish individuals were distinguished from those of eastern, northern and central European descent. These relationships were further defined by inclusion of additional individuals with the same country of origin genotyped with the 300K SNP set (Table 2). Similar results were also observed using a STRUCTURE analysis of the same dataset (Table 2). The results suggest that the difference in numbers of individuals of Irish ancestry was primarily responsible for the major difference in substructure observed in the NARAC cases and controls [23]. Controlling for this aspect of substructure the λ_gc_ in this individual set decreased from 1.15 to 1.07. Since the sample set had a disproportionately large contribution of participants of Irish ancestry we also examined a small set of individuals with nearly proportionate representation of Irish, German, Eastern European, and United Kingdom individuals. Similar to the results on the larger set of individuals, these PCA results showed a west-east gradient (Figure S4). Here however, there was no difference observed between the Irish and UK individuals. Thus, these results further indicate that the number of individuals from each individual group may partially alter relationships among individual groups.

### Principal Component Analysis also Shows Other Aspects of Genomic Architecture

**Figure 5:**
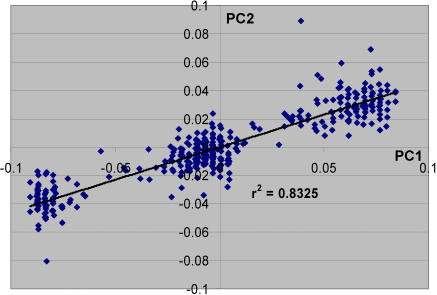
Principal Component Analysis Shows Chromosome 8 Inversion The selected informative SNPs from a 3.8 Mb segment of Chromosome 8 shows the same PC score distribution as the entire SNP set for the second PC in analysis of “northern” European individuals. The graph shows the position of each of 382 tested individuals for the second axis in the PCA using 500K SNPs (ordinate) and the position based on analysis using 20 selected SNPs from the 3.8 Mb segment of Chromosome 8 (abscissa). The 20 selected SNPs were those with the highest I_n_ between the outer groups in an independent dataset separated by a minimum of 50 kb.

Inspection of the second axis of the Northern European subset (see Figure 4A and 4B), also showed an unexpected grouping of individuals on the Y axis into three separate groups. When we ascertained informative SNPs between the top and bottom groups, all of the SNPs with I_n_ values >0.02 were found to be located in a 3.8 Mb segment of human Chromosome 8 (8.135 – 11.936 Mb). This region has been previously shown to contain a common inversion within European populations [24,25]. When only SNPs within this interval were used the distribution of the individuals formed the same grouping of three clusters as found using the entire 500K set (data not shown). As expected two dominant haplotypes (A and B) were ascertained with twenty selected markers with very large I_n_s and described the same three individual groups (AA, AB, and BB) and were highly correlated (r^2^ = 0.83) (Figure 5). Although the λ_gc_ in the entire NARAC case-control dataset is decreased from 1.073 to 1.048 by considering this axis, our analyses indicate that the position of individuals on this axis is almost completely due to this localized inversion. This region is presumably identified by PCA because of the long stretch of linkage disequilibrium caused by the chromosomal inversion.

### Selection of ESAIMs for Northern European Population Studies

Another set of ESAIMs (north-ESAIMs) was ascertained using the results of the first PC scores of the “northern” European only analysis. We selected two disparate sets of individuals comprised of 93 and 132 individuals, by randomly selecting half of the individuals with PC scores one standard deviation above the mean and half of the individuals with PC scores one standard deviation below the mean. We used this procedure to provide both a distribution of allele frequencies in the disparate individual sets as well as maintain a well distributed set of individuals for evaluating the functional performance of the putative north-ESAIMs. These ESAIMs were then selected using the same method (I_n_ values) and criterion (minimum inter-SNP distance = 500 kb). We initially examined the best 1250 north-ESAIMs with 1608 individuals that had not been included in any of the ESAIM selections. Initial evaluation of this north-ESAIM set showed a distortion of the PCA in which the individuals were divided in three groups diagonally across the first two axes. Deletion of markers within the Chromosome 8 inversion (see above) resulted in a set of 1211 SNPs that no longer showed this pattern. This north-ESAIM set distinguished “northern” European individuals in a pattern similar to that observed using the 500K SNP set along the first PC (Figure 4C and see Table S2 for SNP list). The PC scores using these north-ESAIMs in the “northern” European only set correlated with the 500K first PC result; r^2^ = 0.46 (*p* < 10^−15^). Smaller north-ESAIM sets showed dramatically smaller correlations if the individual individual values compared to the 500K PCA (data not shown). Larger panels of SNPs (up to 5000 SNPs) chosen using the same criteria showed similar results to the 1211 ESAIMs.

### Analyses Show Additional Substructure

**Figure 6:**
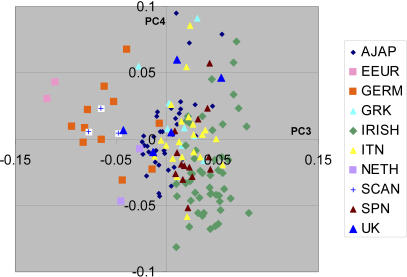
Graphic Display of Principal Components 3 and 4 after Deletion of Chromosome 8 Inversion Results of individuals with 4GP information are shown.

To further explore European substructure we examined additional PCs in the initial all European participant set after excluding the SNPs within the Chromosome 8 inversion. The distribution of individuals in the first two vectors in the entire group did not change. However, the third vector now showed clusters corresponding to population affiliation (Figure 6). However, this PC accounts for only very small amount of the population variation within our different sample sets (see Table 1). Although PC4 showed marginal evidence for clustering by the ANOVA test there was little apparent correlation with self-identified ancestry. Additional PCs did not show evidence for substructure by ANOVA, or a significant split half reliability test.

### Application of ESAIMs to Association Testing

To examine whether ESAIMs could control for European population substructure in association testing the cases and controls from the NARAC RA studies were analyzed for selected SNPs. These analyses were performed using a set of individuals that did not include those used in the ESAIM selections. The SNPs for testing were selected based on our previous results to specifically address the effect of population substructure (Table 3). These included two gene associated SNPs that showed potential false positive results in our association tests, rs1446585 for lactase (LCT), and rs12203592 for interferon regulatory factor 4 (IRF4). In addition, a PTPN22, a TRAF1 and an MHC SNP were included in the testing as positive controls since these SNPs and gene loci are believed to be RA susceptibility genes based on our studies in European populations [23]. As a comparison to the ESAIMs, the same data was analyzed using the entire 500K SNP set with the EIGENSTRAT method.

**Table 3:**
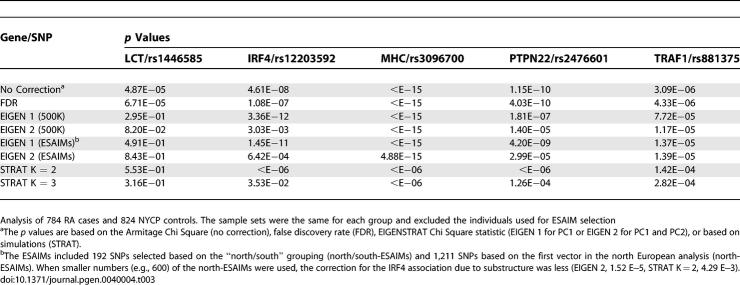
Effect of Population Substructure on Association Testing in Rheumatoid Arthritis Studies

As expected when the EIGENSTRAT analysis was performed using the entire SNP set, this showed strong evidence for association of the PTPN22, TRAF1 and the MHC SNPs. Similarly, when the ESAIMs were applied either using EIGENSTRAT or a method for structured association (STRAT), evidence for the association of these SNPs remained after controlling for population substructure (Table 3). For the LCT SNP in strong linkage disequilibrium with lactose intolerance the evidence for association (*p* < 5E−5) was no longer present when the entire 500K SNPs or ESAIMs were utilized in EIGENSTRAT (*p* > 0.05) or the STRAT analysis (*p* > 0.05).

For IRF4, the association becomes stronger after correcting for the north/south difference (Eigen statistic 1). However, when the second PC is considered the signal is greatly diminished. With the combined ESAIMs (north/south and north), the evidence for association is also greatly diminished by the EIGENSTRAT analysis and eliminated in the STRAT analysis. We examined different sets of ESAIMs including several different combinations of north/south ESAIMs and north-ESAIMs. The results were identical when 192 north/south ESAIMs or 384 north/south ESAIMs were used for the PC1 correction (data not shown). However, as expected based on our PCA results, decreasing the 1211 north-ESAIMs led to poorer PC2 correction and less complete correction of the false positive IRF4 association (see footnote Table 3). Together these results suggest the potential application of ESAIMs in association studies of candidate genes or in replication studies (see discussion).

## Discussion

The current study provides additional insight into European substructure and differences among different ethnic groups that may impact our understanding of the genetics of complex diseases. First, together with our recent report of a whole genome association study for RA in European Americans, this report emphasizes the importance of controlling for substructure in the ascertainment of putative susceptibility associated SNPs. Most notably without an analysis of substructure, IRF4 would appear as a very strong candidate for this disease. However, the large differences in allele frequency for this gene are largely due to the difference in allele frequency among different European subpopulation groups. Furthermore, this difference is accentuated when the northern population subgroup is examined. When only NYCP controls are considered an IRF4 SNP (rs12203592) showed the largest allele frequency difference between the Irish individuals and those individuals of Northern, Central European and Eastern European descent (δ = 0.40, Fst = 0.27). Using an algorithm based on the PCs, EIGENSTRAT, this SNP no longer appears significantly associated with RA. The difference in allele frequency for IRF4 within European populations has recently also been described by the Welcome Trust Case Control Consortium study [26].

The current study extends and complements other studies showing evidence of European substructure. Overall, the current results are consistent with a major north/south (or northwest/southeastern) gradient as the largest difference within European groups confirming both our previous studies and others using up to 10,000 markers and is generally consistent with much earlier studies using classic gene-frequency data [15,18]. The current results differ from previous studies in defining a northern European axis that was critically important in the case control analyses [23]. The relationship between the population groups was consistent when analysis was restricted to “northern” European population groups. As discussed further below, when more disparate populations are examined (including different “southern” populations) these relationships are not as clearly defined (see Figure 2). Thus, differences in these results compared to other studies can in part be attributed to both inclusion of different population groups and perhaps complex relationships reflecting different population origins that includes migration, admixture, and isolation. In addition, the much larger SNP set, 300K compared to maximum of 10K SNPs in previous studies, is also likely to have exposed aspects of substructure not evident in other studies. For PC's >1, comparison of sets of <11K SNPs had much lower correlations with the full 300K SNP set than those random sets with >40K SNPs (Table S3).

Our results also suggests the potential for further definition of more homogeneous population groups for genetic studies that may theoretically decrease both type 1 and type 2 error rates. Geneticists have long recognized that different population groups may provide enhanced opportunities to uncover susceptibility loci based on more limited genetic heterogeneity. For complex genetic diseases some specific studies may focus on particular population groups to enhance the power to find important gene variants. For example, the study of Crohn's disease [#266600] in Ashkenazi Jewish individuals has the advantage of examining a potentially more homogeneous population with a higher frequency of this particular disease than in a mixed European population. This approach is supported by our results suggesting that a very large proportion of this particular ethnic group can be distinguished by analysis of substructure. Moreover, our results provide the ability to further define and restrict this study population by allowing the identification and exclusion of subgroup outliers in association tests in studies of complex genetics in Ashkenazi Jewish populations. In addition, pre-genotyping of potential cases and controls with as few as several hundred north/south-ESAIMs could enable pre-identification of a more homogenous subgroup for WGA or be utilized in candidate SNP replication studies to reduce error rates.

With respect to identification of population substructure there are several limitations in the current study. First, analyses are based on a diverse set of individuals of European descent with variable ancestral contributions from different European countries that is only partially defined. This limits certain conclusions with regards to specific aspects of substructure related to population subgroups. However, we believe that the concordant grouping of the majority of participants with grandparental information provides strong support for the major relationships and differences in these population groups. The overall strong correlation between results using principal components and those using a Bayesian clustering algorithm provide additional confidence in the general results. Second, the PCA is sensitive to differences in the inclusion or exclusion of specific population groups. When the second axis is considered for the entire European group, we observed changes in the country-of-origin order for the northern group with respect to the southern group in subset analyses (Figure 2). We speculate that this observation may reflect the difference in the origins of the additional substructure in the northern group compared to the other elements of substructure in the southern group. This result suggests that overall geographic suggestions of clines based on principal components must be cautiously interpreted. Third, the PCA can be dramatically affected by differences in relatively small genomic regions that may not reflect true population substructure. This is illustrated by our finding that the second axis in the “northern” European analysis (also observed for the third axis in the entire European set) is dependent solely on a <4 Mb segment of Chromosome 8 that carries a common inversion. The effect of such an inversion on PCA is presumably due to a long stretch of linkage disequilibrium that is a result of non-recombination between the inverted and non-inverted chromosomal segments. The genomic distribution of particularly informative SNPs for each PC axis provides one method to inspect whether the apparent differences in substructure are due to a single or very limited number of genomic intervals. For the first two axes of the PCA the particularly informative SNPs, ESAIMs, are widely distributed (Tables S1 and S2). Deletion of subsets of particularly informative markers (e.g. SNPs in lactase and MHC regions) did not change the patterns observed using these ESAIMs for either PC1 or PC2. Since we observed that the Chromosome 8 inversion affected the PCA, we also examined the common European inversion on chromosome 17 [27]. Here, deletion of this chromosomal interval had no effect on the first 10 PCs presumably due to the smaller size of this inversion, 900 kb compared to ∼4Mb for the Chromosome 8 inversion.

An interesting observation in this study is that within the “northern” European population group, individuals of Irish descent showed substantial differences in substructure compared to participants of Scandinavian, Central, and Eastern Europe descent. It also appears that United Kingdom individuals were intermediate between the other non-Irish groups and those of Irish descent further supporting an east/west gradient (Table 2). However, the later observation is based on small numbers of individuals (six 4GP United Kingdom individuals). It is unclear whether these relationships may reflect remnants of early populations including differences in Mesolithic or Neolithic contributions to the Irish population 5,000–6,000 years ago [28], or later Celtic contributions. An extensive Neolithic contribution from the Iberian peninsular is consistent with Irish archeological information but it is unknown whether this population group survived [28,29]. As discussed above, it is difficult to determine the relationship between certain population groups and the suggestion of a cline extending from the Spanish to Irish population is tenuous based on the current data. However, we note that there is modest support for such a cline in both PC2 and PC3 (Figures 2C and 6)

The current study identifies SNPs that are particularly informative for European population substructure (Tables S1 and S2). This includes two SNP sets: one that distinguishes substructure along the “north/south” gradient and the other that distinguishes substructure along a west-east gradient among northern European groups tested. Together these ESAIMs appear to provide good control for subpopulation differences in the NYCP individuals as demonstrated by testing a real dataset using both EIGENSTRAT and structured association methods. Additional studies will be necessary to further optimize ESAIM sets and in particular to determine their efficacy in additional European and European American sample groups that may have different ancestral representation. Finally, it is worth noting that particularly informative ESAIMs may correspond to population selection events and hence also be linked to important biologic processes. The most informative locus for the “north/south” distinction, a lactase gene associated SNP, has been previously noted in this regard [6]. Another strong candidate for selection includes the IRF4 gene that is an important immunologic response regulator [30–33], and ongoing studies are examining these and other genes for evidence of positive selection in different subgroups.

## Methods

### 

#### Populations studied.

For all populations, blood cell samples were obtained from all individuals, according to protocols and informed-consent procedures approved by institutional review boards, and were labeled with an anonymous code number linked only to demographic information.

The first sample set included European Americans of different regional European origins (952 individuals), Italian (6 individuals), and Spanish (14 individuals) individuals. The European American sample set included 894 self identified European American individuals that were recruited as part of the New York Cancer Project (NYCP); a prospective longitudinal study [34]. The European American group also included 38 individuals of Jewish ancestry for which both the country of origin and the Jewish ethnic information for each grandparent were available for each of these individuals. The Italian and Spanish individuals were as previously described [6]. For the European Americans at least partial grandparental information was available for majority of the individuals.

The second sample set included 1255 NYCP individuals and 900 rheumatoid arthritis probands identified as part of multiple studies including NARAC [35], Wichita Rheumatic Disease Data Bank [36], the National Inception Cohort of Rheumatoid Arthritis Patients [36], and the Study Of New Onset Rheumatoid Arthritis [23]. In addition, for one analysis an additional 10 Swedish individuals were included. These individuals were as previously described [6]. Of the 1255 NYCP individuals, 500 overlapped with the first participant set.

Exclusion of individuals with continental admixture: The individuals included in these studies derived from a larger set of European Americans that had been screened for evidence on non-European admixture as described previously [6]. Only individuals with >90% European ancestry by STRUCTURE analysis were included in these studies. A total of 51 NYCP individuals (from 1255) and 31 RA individuals (from 900) were excluded.

#### Genotyping.

Genotyping was performed according to the Illumina Infinium 2 assay manual (Illumina, San Diego), as previously described [37]. The dataset was filtered for individuals with >10% missing genotypes, and SNPs with >10% missing data, Hardy-Weinberg equilibrium (HWE) (*p* < 0.00001) and individual samples for evidence of possible DNA contamination, cryptic family relationships.

#### Statistical analyses.

F_st_ was determined using Genetix software [38] that applies the Weir and Cockerham algorithm [39], and δ was calculated by determining the absolute value of the allele frequency difference between two populations. A measure of informativeness for each SNP (I_n_) was determined using an algorithm previously described [22]. Linkage disequilibrium was examined using the Genetix software [38]. False discovery rate statistics [14] were determined using HelixTree 5.0.2 software (Golden Helix, Bozeman, MT, USA).

Population structure was examined using STRUCTURE v2.1 [21,40]. Each STRUCTURE analysis was performed without any prior population assignment and was performed using 10,000 replicates and 5000 burn-in cycles under the admixture model applying the infer α option with a separate α estimated for each population under the F model (where α is the Dirichlet parameter for degree of admixture). Runs were performed under the λ = 1 option where λ parameterizes the allele frequency prior and is based on the Dirichlet distribution of allele frequencies. A uniform prior distribution of allele frequencies over all loci is used when λ = 1. Structured association was performed using the STRAT software [8] that performs association tests with population structure information that is provided by a prior analysis with STRUCTURE [21].

For initial STRUCTURE analyses we selected random SNPs based on a minimum inter-SNP distance 500 kb; there was no evidence for LD among adjacent markers in each self identified ethnic set (r^2^ < 0.2). The selected sets contained 3500 to 4500 SNPs that were suitable for STRUCTURE analyses. Larger SNP sets have extraordinary computational time requirements for accurate estimates of the parameter values when applied to studies with large sample sizes.

PCA, PCA control for association testing, and determination of the genomic control parameter (λ_gc_) [14] was determined using the EIGENSTRAT statistical package [11]. Several tests were used to assess the significance of PCA. As suggested previously [7], both analysis of variance (ANOVA) and a split half reliability test adjusted by the Spearman-Brown formula [41] were performed. The ANOVA examined the statistical significance of the difference in PC scores among individual groups pre-assigned based on self-identification. The split half reliability test can determine whether independent (non-overlapping) SNP sets provide the same or different results. Unlike ANOVA this test does not rely on correct pre-knowledge of group assignment. For the absence of population structure the null hypothesis is that there will be no correlation in the PCA results. The split half reliability test was performed three times using 1) alternate chromosomes, 2) alternate half chromosomes, and 3) half genome SNP sets. These sets were chosen to eliminate any dependency in each test between the two half datasets based on linkage disequilibrium. Thus, correlation of the independent SNP sets should be due to similar substructure. In addition, the current study also examined whether the distribution of individuals in each principal component (PC) was normally distributed using the Shapiro and Wilk's W-statistic test for normality [42]. In the absence of population structure, the null hypothesis is that the data will be normally distributed.

PCA can be sensitive to quality control issues that can give rise to spurious clustering [43]. Several factors in our design and execution mitigate against this possibility. First the individuals from different ancestry groups and the Irish group in particular were randomly distributed over plates. Furthermore, the genotyping of approximately half the individuals was performed separately. Comparison of the first run and the second run showed very similar results with respect to the distribution of self identified ancestry groups. As indicated in the methods, we used both genotype completeness as well as a loose (*p* < 0.00001) HW exclusion to exclude SNPs with genotype artifacts. Finally, as shown in Table S3, independent random sets (three) showed very strong correlations with the 300K set for PC1 and PC2 (r^2^ all above 0.93).

## Supporting Information

Figure S1 Complete STRUCTURE Results Using 1,441 ESAIMs Selected for North/South Information (909 KB TIF)

Figure S2 Correlation of Individual Substructure Information Using Different Numbers of ESAIMs Informative for “North/South” European Substructure (777 KB TIF)

Figure S3 Graphic Depiction of the “Southern” European Individuals Excluded on the Basis of STRUCTURE Results Using North/South ESAIMs (1.4 MB TIF)

Figure S4 Analysis of European Substructure in 42 Northern European Individuals (1.1 MB TIF)

Table S1 North/South European Substructure Ancestry Informative Markers (147 KB RTF)

Table S2 European Substructure Ancestry Informative Markers Distinguishing Northern European Populations (131 KB RTF)

Table S3 Correlation of Results Using Different Numbers of Random SNPs (53 KB RTF)
