# Supplementary material for: Analysis and Application of European Genetic Substructure Using 300 K SNP Information
Source: PLoS Genet. 2008 Jan 18;4(1):e4. doi: 10.1371/journal.pgen.0040004 (PMC2211544; doi:10.1371/journal.pgen.0040004)
Supplement: Supplementary file 1 [file pgen.0040004.st001.rtf]

Table S1. North/south European Substructure Ancestry Informative Markers^a^						
rs number	Chr	cM^b^	bp	IN	Top 384	Top 192
rs12124147	1	3.359949197	3157488	0.0704398	1	1
rs2012852	1	8.956271161	5475471	0.0295217		
rs201171	1	11.84420167	6585359	0.0238823		
rs6698830	1	14.79035376	8788811	0.0236247		
rs7548659	1	18.53193901	11041705	0.0386892	1	1
rs703804	1	22.2464197	12802503	0.018479		
rs1965402	1	27.2905156	15063705	0.0250388		
rs1005753	1	31.08429882	17190075	0.0225232		
rs4920537	1	36.40147508	18854489	0.0235902		
rs12404648	1	40.6754066	21754104	0.0220498		
rs760607	1	42.89979511	23607959	0.0425441	1	1
rs1981135	1	45.18394514	26131881	0.0407335	1	1
rs4908343	1	46.58760366	27615840	0.0186411		
rs749663	1	48.68526843	29977837	0.0338015	1	
rs669538	1	52.41677121	32380453	0.0275419		
rs6665029	1	55.79896467	34710084	0.0282633		
rs1334804	1	58.02031374	37064337	0.0354169	1	
rs898979	1	60.82568501	38336007	0.0283773		
rs704782	1	63.25983635	39853826	0.0243815		
rs10889750	1	65.63060198	41080342	0.0312527	1	
rs11581921	1	66.76219933	42885023	0.0181616		
rs10493116	1	67.8932813	44199447	0.0148801		
rs4233497	1	68.76684333	45432846	0.0136862		
rs4660344	1	69.51783699	46517242	0.019254		
rs722656	1	71.43591141	48212766	0.0221801		
rs7535374	1	72.21415393	50678918	0.0178489		
rs12075035	1	72.48690246	51735433	0.0191082		
rs1288367	1	74.04641917	53317905	0.0339101	1	
rs213496	1	76.09002906	54581837	0.0381507	1	1
rs17469007	1	78.65715163	56584815	0.0168906		
rs973236	1	82.36808343	58481778	0.0231985		
rs3767996	1	85.03957509	59993733	0.0225677		
rs11207865	1	87.91284495	62099673	0.0310625	1	
rs4915691	1	91.83141096	65579540	0.0340564	1	
rs1566246	1	93.44430847	66957296	0.0460954	1	1
rs10493427	1	95.11944042	67993885	0.0230733		
rs1740909	1	97.30869733	69888266	0.020962		
rs3101336	1	99.44972413	72463206	0.0344699	1	
rs1360244	1	99.71967559	73770945	0.0228367		
rs12732329	1	100.3849632	75322664	0.0200844		
rs544858	1	101.7280551	76437280	0.032158	1	
rs4949662	1	103.2270652	77792296	0.0241573		
rs10493649	1	104.5163977	79920080	0.0473463	1	1
rs12758138	1	105.241609	80942034	0.0317091	1	
rs962249	1	107.16349	82273580	0.0197631		
rs11163752	1	108.0694712	83646461	0.0290567		
rs11161514	1	109.2828401	85217718	0.0236415		
rs4912461	1	110.4568858	86259819	0.028461		
rs10873850	1	111.6762744	87666563	0.0275367		
rs10923099	1	112.9753307	88721286	0.024017		
rs11804087	1	114.5528591	90359715	0.0215706		
rs6660484	1	116.1043043	91884629	0.0469059	1	1
rs497511	1	117.59706	94235134	0.0279661		
rs2968485	1	119.1841148	96581954	0.041456	1	1
rs712886	1	121.1509315	99463539	0.0329034	1	
rs12061503	1	122.7060025	101555275	0.0353462	1	
rs1356394	1	124.1076137	102769291	0.0180602		
rs12046602	1	125.7483425	105371084	0.0338318	1	
rs1419000	1	128.2090099	108394942	0.0274737		
rs731756	1	131.0851271	110573157	0.0439669	1	1
rs11485101	1	135.466089	114214010	0.0189461		
rs10776798	1	137.0895633	115574296	0.0311538	1	
rs544173	1	139.8218746	118012792	0.02552	
rs10923724	1	140.4435511	119258884	0.0229833	
rs1298954	1	142.0719239	143219204	0.0206294	
rs10494257	1	142.7632759	144479385	0.0154162	
rs6587597	1	145.4221426	148333804	0.03313	1
rs12754243	1	146.245401	149498098	0.0274384	
rs7515716	1	146.9560835	150521979	0.0191365	
rs4578216	1	148.0155212	151713794	0.0189365	
rs1778826	1	150.2830982	153351803	0.023736	
rs912572	1	152.0758613	154874699	0.0318948	1
rs4656236	1	154.7650308	156224048	0.0308921	
rs10494362	1	158.7548201	158689377	0.0239054	
rs2841959	1	160.5723298	159789852	0.0306941	
rs1419074	1	165.0963311	161751659	0.0340756	1
rs10753757	1	168.5316821	164114032	0.0152413	
rs1412337	1	170.4219246	165350299	0.0235727	
rs488150	1	172.660274	166883175	0.0458425	1	1
rs12406353	1	174.8523815	168703974	0.0147547		
rs1322780	1	175.9134328	170597243	0.0310913	1	
rs859362	1	177.1986438	172226747	0.0351535	1	
rs6680701	1	178.6606791	174044935	0.0321235	1	
rs10753193	1	180.0037914	176066641	0.0210599		
rs558994	1	182.1306938	178209182	0.0290032		
rs6424913	1	185.1734581	180530534	0.0302557		
rs10911659	1	186.1645657	181636918	0.0171126		
rs2146060	1	186.9430102	182975239	0.0337163	1	
rs4255388	1	188.8595772	185502246	0.0147313		
rs2419063	1	189.5954216	186988492	0.0136448		
rs10921094	1	190.8927964	188846463	0.0182462		
rs1360420	1	193.5430615	191692088	0.0166643		
rs1339951	1	193.8965917	193214014	0.0285156		
rs2784101	1	195.1315816	195489018	0.0360826	1	1
rs2204309	1	196.0579871	196931628	0.0164672		
rs1400875	1	199.6695271	198553100	0.0285791		
rs2494302	1	203.5419339	199976871	0.031464	1	
rs734773	1	206.6614113	202196648	0.0223789		
rs10494878	1	209.0909318	203304204	0.047583	1	1
rs2590691	1	210.5399046	204834195	0.0232256		
rs4844484	1	213.1966289	206226078	0.0168532		
rs4532912	1	214.4590812	207410182	0.0264409		
rs351408	1	215.606186	208829523	0.0247611		
rs12755958	1	218.3911723	211039175	0.0213484		
rs2818964	1	221.1701207	213070843	0.0292717		
rs4373767	1	225.1327316	216148077	0.0279876		
rs2889895	1	225.9678599	217216525	0.0179681		
rs10495177	1	226.9334105	218347393	0.0128835		
rs7517868	1	228.4661611	220558401	0.0221715		
rs7524430	1	230.1621555	223000277	0.0198024		
rs1930903	1	233.0722028	226168221	0.0375023	1	1
rs2275302	1	237.5076661	228897601	0.0337052	1	
rs7546393	1	239.265905	229904086	0.0285156		
rs270505	1	241.0203145	230907654	0.0229324		
rs7555113	1	244.3342697	232094419	0.013898		
rs10802626	1	249.537431	234122878	0.0269435		
rs3748540	1	256.9141903	237019752	0.0302903		
rs10803002	1	260.1923775	238416455	0.0218762		
rs2490400	1	263.1615082	239818684	0.0147725		
rs10924125	1	268.6494234	241714190	0.0227254		
rs4916113	1	274.8534772	244703333	0.0351216	1	
rs4246561	2	3.07479064	1142434	0.0232858		
rs587913	2	9.572700614	3970252	0.0370888	1	1
rs10495532	2	14.01187415	6035514	0.0219702		
rs6723966	2	16.96977883	7196985	0.0312267	1	
rs409169	2	21.57534663	8506682	0.0122388		
rs10929646	2	26.49399876	10335695	0.0344447	1	
rs4260216	2	29.10991481	11486121	0.0418599	1	1
rs779914	2	32.50475045	12651338	0.0163898		
rs4233905	2	34.2968936	13799537	0.020394		
rs2193908	2	35.26662586	15233169	0.0186439		
rs340747	2	38.29191326	16374343	0.0466824	1	1
rs1489688	2	41.01879487	18583881	0.036961	1	1
rs1427547	2	41.7417178	19661604	0.0170784		
rs4665797	2	45.15205971	21581428	0.059436	1	1
rs1438131	2	47.18350591	23418434	0.0331076	1	
rs13032262	2	48.17267438	25557972	0.0341819	1	
rs4450561	2	49.52663084	26565108	0.0240807		
rs10205008	2	50.92937839	28676960	0.0409601	1	1
rs605832	2	55.08417048	31366196	0.0359752	1	
rs7594173	2	55.86215291	32811981	0.0346484	1	
rs17338665	2	57.03580436	33858663	0.0274793		
rs12987048	2	61.2179976	36936364	0.0175244		
rs6729862	2	62.79879675	38054641	0.0257479		
rs2168043	2	64.09223459	39243744	0.0190851		
rs384994	2	64.93765392	40363375	0.0219328		
rs7580660	2	66.14973514	42048439	0.013499		
rs6544718	2	68.77533126	44016576	0.0321417	1	
rs1124787	2	72.07800084	46226586	0.0185865		
rs6713506	2	73.86080674	47949982	0.0312016	1	
rs12621581	2	75.60459249	50102004	0.0197283		
rs903231	2	78.24244434	53606497	0.0189765		
rs7577894	2	79.45755039	55920555	0.0349671	1	
rs10490113	2	82.3745909	59410998	0.0227202		
rs4462771	2	83.67257575	60980940	0.0225531		
rs2075375	2	84.67459413	63192341	0.0651684	1	1
rs1861407	2	85.66973517	64851040	0.0161517		
rs11675929	2	88.01346944	66209259	0.0191391		
rs6715551	2	90.5993272	67947613	0.0277134		
rs6720799	2	92.84320138	70283680	0.0367947	1	1
rs41420	2	96.88520271	72313909	0.0336935	1	
rs13416937	2	98.50900405	74137089	0.0147893		
rs13032535	2	100.4151479	75786972	0.03644	1	1
rs7561233	2	101.3143146	76940978	0.0267583		
rs1370637	2	102.3297539	78252900	0.0204576		
rs6742029	2	105.5758525	80628349	0.0283276		
rs1358192	2	107.8121112	84283293	0.019178		
rs11547160	2	108.9250489	85448073	0.023256		
rs11682587	2	110.3473667	86589154	0.0197959		
rs4129190	2	111.6053841	88312053	0.0202335		
rs11164050	2	112.6995831	95774325	0.0332072	1	
rs11123861	2	112.9045584	97768400	0.0419799	1	1
rs988825	2	113.7850352	99034233	0.0304736		
rs3849381	2	115.0155697	100910337	0.0236209		
rs1041973	2	116.308353	102413986	0.0192903		
rs6739285	2	118.1914885	104815835	0.0490628	1	1
rs1516228	2	120.8716332	107732890	0.0228451		
rs4676286	2	122.497559	109470004	0.019516		
rs1107480	2	123.9397539	111669485	0.0267837		
rs1867828	2	125.3849062	113484851	0.0199469		
rs4490198	2	126.576825	114880689	0.0187081		
rs13425437	2	127.7494316	116550467	0.0308919		
rs4849587	2	128.7533722	118072521	0.0237401		
rs4849760	2	129.2716976	119624284	0.0299827		
rs277554	2	132.0480313	121347614	0.0276485		
rs11692789	2	135.038052	123105106	0.0249083		
rs6739399	2	136.8250675	125132837	0.028502		
rs881944	2	139.3421066	127181878	0.0228254		
rs8179791	2	141.3483284	128773147	0.0192787		
rs1955394	2	142.9213302	130339118	0.0404516	1	1
rs1542604	2	144.0424863	131426777	0.0197849		
rs16829231	2	147.2842792	134365354	0.0384244	1	1
rs6730157	2	149.009446	135740820	0.266153	1	1
rs2117742	2	150.1767336	137882545	0.0753634	1	1
rs11678316	2	153.1030072	141585193	0.0232251		
rs1037266	2	153.8225374	142618009	0.0448467	1	1
rs3768844	2	154.8779526	143630426	0.0200925		
rs13413446	2	156.3119343	145162608	0.021159		
rs10490592	2	157.3321794	146765282	0.0208164		
rs6718445	2	158.1846716	148202457	0.0223289		
rs1975545	2	159.1347008	149442429	0.0180924		
rs330586	2	160.3165289	150826928	0.0267718		
rs6713162	2	161.8362975	152322034	0.0178048		
rs1439638	2	162.8965536	153909634	0.0287694		
rs1867846	2	166.7733016	158012161	0.0309497		
rs3821301	2	167.8322826	159913042	0.0296097		
rs4664403	2	168.8002423	161759222	0.0182755		
rs6752189	2	169.7479027	163975197	0.0699694	1	1
rs6744405	2	170.2584396	165288527	0.0212334		
rs1227131	2	172.2827595	168238305	0.0376472	1	1
rs563694	2	174.1776313	169599578	0.0169323		
rs7574074	2	177.3209866	171374000	0.0173495		
rs6736440	2	180.5258588	174215151	0.0176679		
rs935865	2	182.0694856	175471344	0.029765		
rs6749847	2	183.9009969	177393363	0.0343956	1	
rs934803	2	185.1655792	178516293	0.0207566		
rs4894058	2	185.537313	179564011	0.0175063		
rs4894157	2	185.9906379	180807230	0.0257618		
rs7558428	2	186.4945643	181844060	0.036819	1	1
rs1978650	2	187.9847649	183242647	0.0250571		
rs850903	2	189.3859827	186043389	0.0230847		
rs1201151	2	190.2687252	189549482	0.0198217		
rs9288172	2	190.7890134	191075784	0.0328572	1
rs7571217	2	192.3404169	192153470	0.0157959	
rs10497741	2	194.4470714	193745942	0.0128688	
rs2060798	2	194.5957497	195126236	0.0200753	
rs6434788	2	195.4897271	196278043	0.0271288	
rs6730027	2	196.3645957	197702435	0.0122342	
rs12473502	2	197.624445	199637658	0.0155053	
rs3769823	2	198.8713316	201948501	0.0357473	1
rs10189499	2	200.5084822	203575555	0.0313386	1
rs4675374	2	200.8604193	204628084	0.0197743	
rs2042484	2	204.9085799	208188568	0.019142	
rs288057	2	206.387221	210218217	0.0281142	
rs7425448	2	207.8756214	212384046	0.027456	
rs6435723	2	209.1065791	213406229	0.0170057	
rs1912185	2	210.8052048	215020174	0.0148721	
rs1317763	2	212.2187453	216276161	0.0175925	
rs1978346	2	214.4548643	217386973	0.0276166		
rs6436094	2	218.2628937	219513102	0.0176487		
rs4583451	2	221.4902504	220878782	0.0258764		
rs1955117	2	223.7451585	221896770	0.0310594	1	
rs10189760	2	226.2243317	223669399	0.0423507	1	1
rs2098533	2	227.6206315	225007924	0.0153508		
rs6436553	2	228.8777081	226119390	0.0409355	1	1
rs2396492	2	231.3988785	228412022	0.0256352		
rs6436883	2	235.0650404	230330538	0.0315873	1	
rs1667305	2	238.3790085	232229334	0.0234731		
rs11693862	2	240.4261846	233824586	0.0208674		
rs10929014	2	242.8177801	234855684	0.0216607		
rs4663587	2	246.6218036	236145806	0.0260201		
rs6715815	2	250.9337644	238323938	0.029274		
rs4676403	2	259.0919454	241303666	0.0297767		
rs17047538	3	1.894659484	775606	0.0200049		
rs10510268	3	10.78249791	3539506	0.0430132	1	1
rs3804989	3	14.55707474	4703008	0.0388229	1	1
rs17043611	3	17.5303919	5824483	0.0341999	1	
rs342042	3	19.57579936	6864799	0.035936	1	
rs6802222	3	22.31031314	7927801	0.0190363		
rs7645857	3	27.04541809	9624470	0.0198538		
rs2581204	3	28.5378421	10790485	0.0295857		
rs4547681	3	31.0489209	12918608	0.0276375		
rs2128162	3	33.08165315	14273268	0.0330955	1	
rs10510446	3	36.14887839	16164025	0.0299731		
rs12330375	3	38.64563387	18366482	0.0302234		
rs3916092	3	40.61270281	19921061	0.0454514	1	1
rs11914832	3	42.59016811	21483437	0.0331796	1	
rs4858524	3	45.06377597	23670947	0.0293819		
rs4858669	3	46.98676351	24971212	0.0298339		
rs2618111	3	50.4766435	27886915	0.0178103		
rs1517150	3	52.76759906	29223540	0.0189327	
rs744751	3	55.49501071	30710941	0.0355529	1
rs9814104	3	58.44416238	32784399	0.0202044	
rs10510658	3	59.84803137	34197193	0.0133977	
rs285327	3	61.37288646	36289024	0.0320544	1
rs11716902	3	64.15214817	38933014	0.0197682	
rs408499	3	65.28669382	41068579	0.0240613	
rs897		3	66.97579794	42553307	0.0156102	
rs1013758	3	67.90359931	43601379	0.0152911	
rs2118749	3	69.27552583	45547261	0.0277355	
rs2291903	3	70.37380801	47692630	0.0127763	
rs3172494	3	70.60144606	48706491	0.0154847	
rs2282751	3	70.88334677	50266789	0.0214684	
rs4416372	3	71.00081465	51916825	0.0204445	
rs7427716	3	73.34511914	54314616	0.0272681	
rs7641315	3	77.2616333	55962863	0.0266965	
rs11544593	3	78.41847956	57278724	0.037625	1	1
rs6773085	3	79.97305223	59447213	0.0345693	1	
rs11130841	3	84.0512912	61378028	0.033629	1	
rs924067	3	87.2402854	63619734	0.0146312		
rs6795735	3	89.01547586	64680405	0.0337705	1	
rs182041	3	90.81580466	65885258	0.0126785		
rs2054956	3	92.77708218	67433729	0.0321825	1	
rs6787753	3	93.69739751	69078627	0.0133171		
rs805486	3	94.9427987	70512999	0.0195885		
rs3749430	3	97.88036254	72231809	0.030945		
rs9310279	3	101.7956444	73793635	0.0372439	1	1
rs11706032	3	103.6810929	75816778	0.0211176		
rs1920552	3	105.9390737	77930481	0.0140093		
rs6766522	3	107.2256314	79160577	0.0261371		
rs6785616	3	107.7138763	81711666	0.0170324		
rs11127782	3	108.3519424	82774509	0.0161465		
rs11127853	3	108.5698035	84711551	0.0227017		
rs9284813	3	109.2332613	87234859	0.0242461		
rs1495824	3	109.9514449	88816348	0.027057		
rs6551458	3	110.1026169	90355016	0.0379391	1	1
rs7630043	3	110.3884258	96097436	0.0432374	1	1
rs11713687	3	110.4719641	97228391	0.027777		
rs9289584	3	111.4162957	99583791	0.0312947	1	
rs7613565	3	111.8934751	100850559	0.0233122		
rs1972991	3	112.2714648	102187236	0.0309177		
rs7633625	3	115.0085955	106552546	0.0187299		
rs6808795	3	117.6017571	109023404	0.0233077		
rs4535234	3	118.7836989	110097182	0.0318292	1	
rs2399399	3	120.1065118	113120594	0.0193764		
rs2062834	3	121.9133186	115432817	0.0309116		
rs7634702	3	122.9630142	116811864	0.0336037	1	
rs1462843	3	125.0529484	118428943	0.0288449		
rs1733302	3	126.9726092	121636871	0.019105	
rs7624614	3	128.8915627	123786619	0.0280076	
rs6800965	3	131.0762419	125746571	0.0174274	
rs4679115	3	133.2171355	127479896	0.0208842	
rs1683804	3	136.2278077	130099023	0.0217181	
rs16827446	3	137.5003797	131614900	0.0180227	
rs6808429	3	138.5252111	132772989	0.0244133	
rs4384926	3	142.6812422	135477763	0.0272452	
rs864800	3	143.8046341	136526412	0.0187276	
rs4678297	3	144.2929025	138389809	0.0326839	1
rs13073891	3	146.3371631	141623140	0.025828	
rs9821235	3	148.0862944	142911401	0.0193028	
rs9839394	3	149.9522395	144434536	0.0342844	1
rs197858	3	151.6933118	145555370	0.0219922	
rs9834043	3	152.4550526	146853100	0.0227352	
rs6788109	3	153.8342171	148871451	0.0184848	
rs7627651	3	158.3232881	151404783	0.0290468		
rs1491975	3	160.3532136	152665895	0.028883		
rs355788	3	162.7555263	155483314	0.0256943		
rs7639767	3	164.670803	158618297	0.0206049		
rs1714521	3	164.9865901	159767563	0.0180333		
rs6762377	3	166.1654555	161323330	0.0286341		
rs9865618	3	166.7136428	163165416	0.0206696		
rs10936383	3	167.3101868	165187391	0.0217406		
rs9851453	3	168.0780743	167404518	0.0149555		
rs9815034	3	168.9110901	168980146	0.0232695		
rs7613341	3	170.720124	170673609	0.0190265		
rs6787400	3	171.6927852	171840090	0.0186639		
rs7620616	3	173.8084188	173036498	0.0298719		
rs635255	3	177.742674	174681061	0.0249852		
rs10513729	3	179.710253	176163949	0.0364454	1	1
rs4857700	3	183.3493826	178711196	0.0154436		
rs7636818	3	184.6086231	180504817	0.03643	1	1
rs12635682	3	185.5078383	182006810	0.0322353	1	
rs10937074	3	186.2131602	183120035	0.0219276		
rs4859259	3	187.7370438	184147249	0.0374279	1	1
rs9872799	3	190.906903	185911605	0.0225715		
rs9867846	3	192.3966691	187229592	0.0260332		
rs6775595	3	203.1360805	190616279	0.0339598	1	
rs10513852	3	204.5785599	191650235	0.0294138		
rs4686599	3	208.5559488	193327814	0.0236964		
rs7642439	3	210.7515198	194432684	0.0194461		
rs2410840	3	216.7277739	196464350	0.0273165		
rs569330	3	219.6690146	198205623	0.0243011		
rs1564282	4	1.08233546	842143	0.0262337		
rs1203766	4	3.135502229	2403488	0.0130652		
rs11732375	4	4.66140287	3433932	0.0194889		
rs7663990	4	7.483917594	4872556	0.0261532		
rs959190	4	23.13726349	10549010	0.0270057		
rs1019661	4	27.40651399	13864860	0.0286441		
rs10001565	4	29.86389494	15398842	0.015737		
rs6449375	4	34.59954998	18068888	0.0370923	1	1
rs3846382	4	35.03835064	19286208	0.0248846		
rs13132286	4	36.93477331	21039910	0.035932	1	
rs1395221	4	40.02233052	24303172	0.020627		
rs6831024	4	43.91001105	25539644	0.0356753	1	
rs4692338	4	48.14357339	28368120	0.016805		
rs6448770	4	51.34713718	31073179	0.0400476	1	1
rs9884706	4	52.83388626	32422156	0.0370375	1	1
rs7664105	4	53.40117274	33758316	0.0159046		
rs1655069	4	54.41414409	35746555	0.030208		
rs4832844	4	55.58685197	36770917	0.0159273		
rs10008492	4	57.93656727	38588286	0.0773511	1	1
rs12186184	4	62.46250055	41138358	0.042525	1	1
rs2120041	4	64.15666649	43108899	0.0232783		
rs1389037	4	65.33714509	45635821	0.0344543	1	
rs11934749	4	66.06707626	47507992	0.0233408		
rs6832891	4	68.29447904	54897572	0.0349493	1	
rs10031105	4	70.56751309	56450800	0.0146689		
rs10222715	4	72.54791939	57455088	0.0153902		
rs4865353	4	74.30015808	59406517	0.0234671		
rs2124236	4	75.38940144	61535122	0.0257814		
rs6843214	4	76.44767264	63183069	0.0257791		
rs6855459	4	77.09102728	64233143	0.0118205		
rs1440949	4	77.94611274	65712326	0.0262691		
rs11724259	4	79.21715462	67988353	0.0240128		
rs1109501	4	80.9305082	71510250	0.061827	1	1
rs4261956	4	82.07671672	72706899	0.033882	1	
rs11722134	4	82.82702599	73921383	0.0134859		
rs3923243	4	84.93671811	75936632	0.0512814	1	1
rs3733242	4	85.9022641	78032684	0.0258031		
rs2055493	4	87.2140868	79339098	0.0292497		
rs1455313	4	88.13401667	80479073	0.0313902	1	
rs10516652	4	89.42284513	82524083	0.0296209		
rs6820697	4	91.89325463	85157654	0.0446565	1	1
rs7654494	4	93.19449028	86668104	0.0198704		
rs2869703	4	95.53721049	88983457	0.0241399		
rs1343921	4	97.58502961	90304640	0.0422728	1	1
rs1355993	4	99.31415651	92018923	0.0159792		
rs1485989	4	100.1911558	93116014	0.018523		
rs10030884	4	100.9755634	94625806	0.0201347		
rs2214395	4	102.0346047	96112018	0.024673		
rs13349989	4	103.0682166	97728559	0.0464386	1	1
rs7669241	4	105.4654041	100788102	0.0371795	1	1
rs2850971	4	106.7730514	102541804	0.0323464	1	
rs13152048	4	107.3468784	103633018	0.0159479		
rs7682418	4	109.1786961	105786021	0.0139212		
rs4235415	4	109.9073009	106951454	0.02022		
rs1056079	4	111.0706483	108580375	0.0165273		
rs2194860	4	112.6670698	110306115	0.046987	1	1
rs1448817	4	114.6502423	111998657	0.0353719	1	
rs1557815	4	118.4083026	115053977	0.033793	1	
rs4574434	4	118.7916458	116097032	0.0370477	1	1
rs1541745	4	120.7772645	117848736	0.0458366	1	1
rs4128688	4	121.4380763	119146195	0.0338168	1	
rs10518336	4	122.2758592	120880537	0.0286925		
rs692157	4	123.4799867	122740439	0.0468934	1	1
rs13115814	4	124.5646882	124903113	0.0245235		
rs3956574	4	125.605105	126396940	0.0256695		
rs937923	4	126.3766527	127763996	0.0182462		
rs1602084	4	127.2069464	129201085	0.0154065		
rs2391576	4	128.2637956	131285066	0.0208658		
rs2419533	4	130.5883136	133947119	0.0247472		
rs1390009	4	131.5970928	135289936	0.04041	1	1
rs1451118	4	132.8110909	137464571	0.0205453		
rs10519410	4	133.6862557	138492186	0.0617087	1	1
rs706343	4	136.2927585	140906318	0.0275132		
rs3775605	4	139.5832708	143325686	0.0238809		
rs1866179	4	140.904323	146742380	0.0237325		
rs1507500	4	143.0348223	148351307	0.041419	1	1
rs6852150	4	144.9328643	151176267	0.0195607		
rs7663659	4	145.2392912	152532773	0.0184113		
rs2054758	4	146.2061109	153762817	0.0296767		
rs11935573	4	149.775942	155599177	0.018609		
rs1563806	4	150.7500342	157050423	0.0266597		
rs10517660	4	151.8248834	158200001	0.0333114	1	
rs7678673	4	153.6884756	160496864	0.0295799		
rs1492468	4	155.7598564	163387617	0.0314439	1	
rs1430975	4	158.6018825	165466669	0.0362301	1	1
rs703887	4	161.2102323	167494113	0.0235872		
rs10517954	4	162.3013512	168985788	0.0282633		
rs3811813	4	163.9567573	170412302	0.019364		
rs744724	4	168.1662498	174834553	0.018002		
rs11730565	4	170.0687709	176701588	0.0325881	1	
rs1011851	4	172.5667812	178545485	0.0180758		
rs7673020	4	177.8109567	181337434	0.0226414		
rs1454711	4	180.3933779	182486507	0.0279448		
rs1435442	4	188.0128725	185192351	0.0490033	1	1
rs4241802	4	193.072759	186776780	0.0319556	1	
rs2165666	4	197.560149	187836855	0.0240055		
rs10032784	4	204.6063124	190643908	0.045157	1	1
rs2736122	5	1.580479002	1310621	0.0517431	1	1
rs698147	5	9.451835427	3566485	0.0256114		
rs26880	5	12.20584102	4958892	0.0305379		
rs982248	5	15.95558529	5996182	0.0228591
rs7713650	5	19.14465224	7222448	0.0149169
rs4702561	5	22.10594298	8637086	0.0283598
rs6555619	5	25.15006862	9949146	0.0301793
rs31887	5	29.43923831	11476592	0.0202778
rs10513144	5	31.48436526	13686968	0.0243957
rs4235556	5	34.81605167	15791017	0.0207245
rs1872004	5	37.15966964	16860786	0.0250249
rs10472159	5	39.10206342	18767817	0.0226989
rs1155040	5	39.89779319	19849864	0.0137791
rs17375683	5	42.63760433	23277554	0.0207569
rs7714385	5	44.02388748	24536402	0.0143508
rs1565873	5	45.84882999	26410688	0.0151518
rs16896876	5	47.00074376	27559097	0.0263893
rs182778	5	48.30645574	29586161	0.0180418
rs7381059	5	50.69766867	31221471	0.0268265
rs1051489	5	53.82752845	32436023	0.0204172	
rs299596	5	56.02613755	34067771	0.020168	
rs9292587	5	57.54278544	35400545	0.0148254	
rs4418129	5	58.48568623	36433781	0.017789	
rs2910725	5	59.48979002	37772804	0.0173028	
rs696737	5	63.69972674	39231180	0.0200753	
rs1553578	5	64.966099	40704941	0.0181001	
rs10512806	5	66.33009112	42874313	0.0217334	
rs6875400	5	66.63336557	43898451	0.0134489	
rs13340341	5	67.27767321	45896693	0.0185111	
rs1039797	5	67.3768882	49687629	0.01393	
rs4865695	5	67.47826783	51234866	0.0176828	
rs2269225	5	70.09956182	54311861	0.0191222	
rs12523475	5	71.36183841	55692321	0.0166512	
rs7718757	5	73.13291564	56965873	0.0357908	1
rs1867730	5	74.51987545	58078758	0.0205415	
rs1533019	5	75.4319688	59343570	0.0339955	1	
rs10054295	5	77.08457926	63071679	0.0301162		
rs29460	5	78.50712662	65570159	0.035068	1	
rs2888306	5	80.39556314	67448240	0.0240983		
rs464923	5	84.27607873	72213646	0.0322937	1	
rs2962622	5	85.88729839	73584027	0.0232695		
rs1001935	5	89.08400565	75075936	0.0322283	1	
rs2279095	5	91.93314957	76745182	0.0344958	1	
rs234688	5	93.76360302	78235210	0.0247222		
rs1373967	5	96.37139356	80252312	0.0460914	1	1
rs173686	5	100.284274	82847256	0.0327049	1	
rs6452576	5	101.4326713	84017558	0.0173812		
rs2974061	5	101.8225635	85262920	0.0197915		
rs6452788	5	103.0204612	87748669	0.0352064	1	
rs1852723	5	103.4930677	88796267	0.0180496		
rs2973442	5	105.0133608	90457972	0.0158246		
rs7725232	5	106.0048241	92600649	0.021993		
rs251083	5	107.5381789	94549621	0.0174804		
rs9127	5	108.9647622	96397921	0.0256114		
rs10515276	5	110.043777	97864694	0.0205543		
rs380360	5	111.4357357	99972178	0.0263463		
rs174015	5	111.9608086	102159866	0.0362067	1	1
rs12514674	5	114.9304238	107186986	0.0295551		
rs4339398	5	116.2117521	109381880	0.0255821		
rs10478046	5	117.4566054	110741382	0.0331056	1	
rs13358140	5	118.2694711	112044477	0.0176085		
rs13186060	5	119.1660191	113235512	0.0295758		
rs10519406	5	120.0125083	114719186	0.0244162		
rs13356416	5	123.031995	116466982	0.0267498		
rs10056223	5	124.666621	117798762	0.0248439		
rs162307	5	125.1057072	119110854	0.0187129		
rs3909319	5	125.6608815	120731816	0.0236836		
rs10519723	5	127.2874376	122739540	0.0165249		
rs6863510	5	128.8843272	124212446	0.0366282	1	1
rs1549189	5	130.4196532	125716195	0.0166068		
rs3909885	5	131.4701987	126946403	0.0282896		
rs6866231	5	133.0481222	128831244	0.0363169	1	1
rs12513768	5	133.4116301	130644691	0.0287535		
rs4958148	5	135.3877269	133100424	0.0260175		
rs346650	5	138.2616981	135752820	0.042809	1	1
rs1528961	5	138.8784019	136821047	0.0377995	1	1
rs3756675	5	139.4794804	139219077	0.024394		
rs918558	5	140.6289243	140851133	0.0253885		
rs7721458	5	144.9506945	142843433	0.0260783		
rs10515553	5	146.9583218	145019135	0.0239731		
rs7707803	5	150.3539166	147368427	0.0200925		
rs3776082	5	153.4933698	149524238	0.0308639		
rs2615152	5	158.4660891	152676300	0.0229406		
rs646289	5	160.0231542	154581940	0.0188583		
rs905788	5	160.9919652	155794803	0.0249835		
rs1650576	5	161.9340732	157587622	0.0269508		
rs7380834	5	162.6959887	158668511	0.0138634		
rs1038704	5	165.0361997	160483665	0.0271409		
rs2964356	5	166.6777738	162559574	0.0185918		
rs1422680	5	169.3709128	164598962	0.0302787		
rs4283811	5	174.4844736	167102662	0.0257278		
rs10516061	5	177.9408681	168532207	0.0244329		
rs10516076	5	181.3425673	169828668	0.0206853		
rs171718	5	183.8879666	171075771	0.0329748	1	
rs4868204	5	186.9023219	172137235	0.0455824	1	1
rs2135053	5	193.8543462	174398116	0.0327931	1	
rs7727897	5	198.6258071	176979615	0.0674604	1	1
rs248327	5	204.0270464	179310256	0.0386077	1	1
rs1933652	6	0	686043	0.0345906	1	
rs2326106	6	8.053744176	2934581	0.0336941	1	
rs6939368	6	12.24509937	4628535	0.0157108		
rs4960257	6	17.76117083	6810243	0.0383857	1	1
rs2876167	6	22.6749881	9589358	0.0376796	1	1
rs12200932	6	26.23763645	11214821	0.0181071		
rs12662518	6	28.79851076	12327771	0.0157643		
rs1992387	6	30.90340031	13539293	0.0440065	1	1
rs2237148	6	34.68413414	15520143	0.0326492	1	
rs7763768	6	37.5486617	17037691	0.0386423	1	1
rs9366309	6	41.43045334	19785803	0.0264279		
rs1928168	6	44.77957861	22125717	0.0267284		
rs9379502	6	46.57464099	23466218	0.0242157		
rs6918101	6	49.52409647	25135267	0.0448427	1	1
rs13194984	6	50.12120191	26608542	0.0285156		
rs13214703	6	50.63902259	28049366	0.0267237		
rs2747454	6	51.00569229	29763092	0.0268412		
rs12660883	6	51.26545049	30872399	0.036655	1	1
rs382259	6	51.73450767	32317005	0.128836	1	1
rs2076173	6	55.15989038	35550375	0.0548773	1	1
rs804847	6	56.90175587	37655491	0.0318859	1	
rs10023	6	57.97331684	39180012	0.019301		
rs9357342	6	60.96305802	40905214	0.0307217		
rs7755400	6	64.35965015	42305894	0.0229338		
rs4562138	6	67.44490605	44234785	0.020207		
rs12524885	6	70.24930893	45944047	0.0312722	1	
rs13212023	6	73.07642756	47754707	0.0215587		
rs12191923	6	73.64847065	49968341	0.0178556		
rs788510	6	74.99315285	51813583	0.0251531		
rs1883632	6	77.87055877	53888391	0.0196333		
rs9296835	6	79.84909889	56229526	0.0216475		
rs721423	6	80.60579447	63092726	0.0185096		
rs2055172	6	81.45639995	65541479	0.020853		
rs9342597	6	82.55035662	67682902	0.0232155		
rs701662	6	83.30720382	68946749	0.0238983		
rs3806033	6	83.96302317	70885057	0.0187172		
rs6914937	6	85.6076597	72036050	0.0261497		
rs12212214	6	87.90152133	74757702	0.0229585		
rs2046500	6	88.93679325	77054123	0.0166431		
rs12211608	6	89.46091809	78275224	0.021993		
rs2057155	6	90.61443129	80505112	0.029879		
rs12207462	6	91.56085788	82512770	0.0238226		
rs1932215	6	92.30034571	83616128	0.0229768		
rs10943994	6	92.87491223	85253049	0.0257728		
rs9359682	6	93.34391201	86953487	0.0155897		
rs6454694	6	94.01062232	89230477	0.0226904		
rs4610536	6	99.14325863	91971586	0.036113	1	1
rs6908465	6	100.0286683	93500050	0.0264582		
rs746810	6	101.6878558	96596810	0.020803		
rs551185	6	104.1292481	100213044	0.0234924	
rs9404063	6	104.978654	101493770	0.0161098	
rs2506756	6	106.7914923	104099986	0.0309779	
rs6908605	6	109.976771	106999734	0.0201123	
rs539630	6	112.5696872	108717334	0.0217375	
rs9320326	6	114.7285781	110763835	0.023693	
rs10457239	6	115.8339913	111916489	0.0266622	
rs4947199	6	117.074139	113120174	0.0235107	
rs352092	6	117.7434549	114347378	0.0203853	
rs638473	6	118.610424	117306516	0.0339215	1
rs7738597	6	119.0760479	118369989	0.0142373	
rs4946395	6	119.5240887	119467668	0.0145222	
rs11750983	6	120.1542864	121570625	0.0172485	
rs13200297	6	122.3041346	123293002	0.0139259	
rs802228	6	124.5250374	124568348	0.0214556	
rs6906366	6	126.7264844	126325906	0.0150371	
rs802726	6	127.7049814	128323554	0.0295135		
rs2451688	6	128.7322628	129688258	0.0336897	1	
rs6940184	6	131.0312185	132299137	0.0306738		
rs9389124	6	133.3203401	134355478	0.0350081	1	
rs7753036	6	142.354476	138953540	0.0364485	1	1
rs4896663	6	146.4311518	143874819	0.0316347	1	
rs1406045	6	146.974569	145046104	0.0302782		
rs17551120	6	148.3898981	147275677	0.0454016	1	1
rs12182899	6	151.0296594	148982366	0.0195703		
rs9322206	6	152.6923157	150004310	0.0176826		
rs11753794	6	155.2230715	151510434	0.0257271		
rs11756366	6	160.7133058	153943708	0.0340434	1	
rs2352423	6	163.7489903	155632343	0.0180069		
rs6557486	6	165.1774578	156676103	0.0178819		
rs590906	6	166.6685803	158088753	0.0251008		
rs2171209	6	167.9439017	159153971	0.0170248		
rs9457827	6	169.9923285	160474067	0.021598		
rs577876	6	171.7980295	161893566	0.02301		
rs11968371	6	175.0187328	163262364	0.0273395		
rs12205431	6	176.2824497	164323472	0.0310255		
rs10428822	6	180.5626081	166574176	0.0403667	1	1
rs9364195	6	187.1142538	169228307	0.0308939		
rs12201680	6	190.6733285	170744099	0.0168526		
rs4281072	7	0.215228418	149266	0.0141512		
rs10950641	7	5.610562355	2107627	0.0336897	1	
rs13240660	7	8.591289264	4038161	0.0148272		
rs4724793	7	12.55863492	6088006	0.0262369		
rs2041362	7	15.56511429	7817723	0.0326492	1	
rs6463875	7	18.49524068	8901481	0.0211692		
rs2189947	7	20.81960525	10092254	0.0459737	1	1
rs739773	7	22.58286435	11167850	0.0268585		
rs10267453	7	26.77046326	13906545	0.0355969	1	
rs697518	7	30.43640964	16532348	0.0432041	1	1
rs10486293	7	32.60707398	18409035	0.0177751		
rs2529015	7	34.80646309	20099826	0.0379692	1	1
rs2905308	7	38.8482076	22421434	0.0295404		
rs10951140	7	42.69604293	26304960	0.0491403	1	1
rs1362352	7	47.85758621	29368014	0.0238717		
rs2302019	7	50.36975394	30776663	0.0218053		
rs6947433	7	52.46522248	31949671	0.0265009		
rs4723307	7	53.80182382	33481877	0.0262112		
rs1003549	7	54.63213991	35068731	0.0315284	1	
rs3801305	7	56.76974186	36254116	0.016232		
rs886765	7	61.93997755	40457092	0.0123162		
rs2214875	7	63.43194291	41566283	0.0255691		
rs12530692	7	64.89788453	43052088	0.0234117		
rs3735477	7	66.49326629	44487752	0.02088		
rs4724556	7	69.45681788	47079209	0.0278743		
rs6948541	7	70.54648285	48174237	0.013571	
rs933360	7	72.71666676	50532454	0.0315459	1
rs17135491	7	75.3648483	52879809	0.0329553	1
rs17564002	7	76.13652377	54570046	0.0282224	
rs4870612	7	78.14476362	57286821	0.0159216	
rs11768782	7	78.7244362	63410316	0.0163781	
rs313830	7	78.9242789	64996081	0.0121493	
rs4718701	7	79.54301639	66959951	0.0261242	
rs7811944	7	81.72463092	68168706	0.0188646	
rs4268007	7	82.96164	69966427	0.0250867	
rs4717112	7	85.7451169	72710068	0.0148957	
rs7804806	7	87.89832025	75240333	0.0190395	
rs4020771	7	91.3690703	78422048	0.0333332	1
rs10258421	7	92.71977076	79776493	0.024663	
rs2075023	7	94.33202731	81224904	0.0253133	
rs7789583	7	96.25290603	82610444	0.0243619	
rs2373124	7	98.8105542	85474923	0.0262564		
rs6949448	7	99.37954264	86786465	0.0249865		
rs6943877	7	100.0651027	88236153	0.014123		
rs7791143	7	100.9897729	89331915	0.0528909	1	1
rs2974129	7	102.5774123	90943812	0.0205736		
rs7810211	7	103.4739598	92435480	0.0287671		
rs6465459	7	105.847938	94484227	0.0195892		
rs1207692	7	106.7467145	96223291	0.0180642		
rs6465748	7	109.1013872	98505958	0.0241188		
rs12539172	7	109.9803551	99736446	0.016043		
rs2410922	7	111.2209045	101220075	0.0159321		
rs802787	7	112.6878141	103143675	0.0298524		
rs2727751	7	115.5025991	105202552	0.024878		
rs4730287	7	118.0428871	107364575	0.0435577	1	1
rs6951925	7	118.663991	108395575	0.0281312		
rs7781715	7	120.0315018	110307171	0.0464007	1	1
rs1404228	7	121.0377599	112644739	0.0297401		
rs12705973	7	121.6813375	113907150	0.038253	1	1
rs6466573	7	122.6171446	115542965	0.0293515		
rs9640779	7	123.7295628	117276052	0.0184272		
rs2896309	7	125.5541359	121025905	0.024394		
rs2079886	7	127.0973551	123452557	0.0125791		
rs1346364	7	127.8570454	124524708	0.0155458		
rs12706793	7	128.9959509	126666091	0.0348355	1	
rs1472147	7	130.0359607	128097494	0.0124698		
rs1593306	7	135.911972	131071218	0.0404275	1	1
rs6952869	7	138.8151825	133167042	0.0210714		
rs11543821	7	139.0674181	134291754	0.0225711		
rs225137	7	142.0905229	136404161	0.0163651		
rs11763343	7	145.1487631	137419441	0.014779		
rs2355787	7	147.7853626	138547183	0.0191105		
rs11761774	7	151.0757231	141314653	0.0440837	1	1
rs1973815	7	152.9044583	144168025	0.02202	
rs4571660	7	157.7041214	147163109	0.0346688	1
rs7795021	7	161.6559353	149519133	0.0317941	1
rs7806048	7	165.5917628	151016399	0.0312236	1
rs768982	7	171.7881676	152983269	0.0219728	
rs9769506	7	177.7529856	154537503	0.0195885	
rs7780536	7	181.3806513	155543440	0.0202875	
rs6459830	7	189.4116091	157334204	0.0175038	
rs3860880	8	0	799497	0.0154195	
rs9644331	8	4.182045823	2490274	0.0262106	
rs10101380	8	10.93692955	4992467	0.0245782	
rs17381669	8	14.24323506	6107334	0.0176247	
rs17150066	8	18.50115996	9347921	0.0307112	
rs4367597	8	21.55001689	11830938	0.0242366	
rs2946513	8	24.15062644	12911168	0.023661	
rs4416821	8	26.47909621	15604101	0.0238957	
rs1548353	8	29.00535809	17211136	0.0366956	1	1
rs6586906	8	34.65465733	20222955	0.0260836		
rs4565458	8	38.7006493	22210078	0.0371191	1	1
rs920300	8	41.78431424	23610510	0.0235297		
rs12548107	8	46.1570654	27113796	0.0521345	1	1
rs6996714	8	48.27073619	28510366	0.0199296		
rs4352803	8	50.76945239	29937093	0.0150512		
rs4987236	8	52.47108178	31041477	0.0122615		
rs10503904	8	53.1837962	32284229	0.0224036		
rs13273386	8	55.03379573	35765533	0.0356783	1	
rs12676965	8	55.84239157	37800584	0.0195924		
rs6474513	8	57.26967736	38923383	0.0142611		
rs1584991	8	58.39940252	40218904	0.0167959		
rs1127379	8	59.9412752	41240437	0.0319789	1	
rs7461363	8	61.18565754	43506631	0.0230638		
rs10109984	8	62.17083262	48966228	0.0507649	1	1
rs1480391	8	62.74114655	50270973	0.0293736		
rs7830163	8	65.05171402	53240995	0.0342764	1	
rs6993747	8	66.49414712	54343468	0.0361212	1	1
rs997855	8	68.09569964	56222422	0.0296465		
rs4316149	8	68.9900302	57695801	0.0279298		
rs4738613	8	69.8877944	58815888	0.0138815		
rs7827918	8	72.05056642	60640671	0.048329	1	1
rs7820685	8	74.74434785	62478963	0.0182126		
rs2061133	8	75.1162813	63992920	0.0136338		
rs3110289	8	75.93310523	65509490	0.0196941		
rs4737912	8	78.81300042	69570691	0.0229511		
rs2380646	8	82.02456244	71521018	0.031917	1	
rs4634634	8	87.03748779	74431380	0.0385856	1	1
rs10504595	8	88.63799988	76738610	0.0264724		
rs11780821	8	89.66399707	79270073	0.0311868	1	
rs11784678	8	89.86510016	80450772	0.0384143	1	1
rs1026804	8	91.06548019	81680875	0.0508262	1	1
rs7845376	8	93.17182353	83385332	0.0169282		
rs9643769	8	93.61249866	84473411	0.0222097		
rs1369938	8	94.49768974	86627957	0.0153009		
rs17623943	8	95.13164436	88186962	0.0268563		
rs10092877	8	95.94199976	89892791	0.0243666		
rs425140	8	96.4429661	90933795	0.0146651		
rs7838815	8	97.15798368	92492120	0.0197052		
rs3735912	8	97.72841127	93544313	0.0280661		
rs4304300	8	98.8901711	94674550	0.0177202		
rs4734283	8	100.0429627	95697990	0.0165892		
rs2331465	8	103.2863127	98497948	0.0231731		
rs921313	8	105.0387163	100893726	0.0136022		
rs1470517	8	107.6519397	102224589	0.0273848		
rs17186961	8	110.5022748	103699204	0.0181739		
rs1841316	8	112.2637695	105911442	0.0449208	1	1
rs2022970	8	113.19625	107479243	0.0304941		
rs16876241	8	113.7559594	108489864	0.0226351		
rs2980618	8	115.0287995	110371174	0.0222752		
rs17652171	8	116.2089614	113731759	0.018331		
rs1257824	8	116.6810169	114827379	0.017925		
rs4876363	8	118.2935798	117705510	0.0221583		
rs3103991	8	119.7364624	119955272	0.0229356		
rs4871195	8	122.5750449	122567434	0.0407003	1	1
rs5009958	8	124.5409446	123745694	0.0236822		
rs7012891	8	129.6698969	126583858	0.0256988		
rs7833560	8	134.2765823	128141201	0.022608		
rs10505517	8	138.2159444	129547193	0.0268465		
rs3829032	8	140.6181669	131902218	0.0216964		
rs4736413	8	142.9725976	133395104	0.0394913	1	1
rs2958830	8	146.4896279	134718528	0.0266486		
rs11166873	8	147.527284	135999906	0.023214		
rs868421	8	151.2430948	138668249	0.0172374		
rs951920	8	154.5331251	140079282	0.0183715		
rs4961381	8	160.7651312	142473737	0.0248316		
rs369051	8	164.3777612	144619949	0.0195769		
rs11988651	8	165.7139099	145789590	0.0151887		
rs9298675	9	1.855544263	1242572	0.0248204		
rs4740717	9	7.005076485	3173137	0.0293972		
rs396861	9	11.70863284	4733626	0.03247	1	
rs10758823	9	15.10772433	7078043	0.0337033	1	
rs7043322	9	18.17482358	8231845	0.0238297		
rs1408125	9	21.39042294	9727201	0.0295404		
rs2382371	9	26.29643541	12770295	0.0230444		
rs3920568	9	28.13046443	14026242	0.0260529		
rs566820	9	31.82560843	15182720	0.031529	1	
rs10962589	9	35.26810276	16773380	0.0446638	1	1
rs2840790	9	36.92922825	17959519	0.0312966	1	
rs4468024	9	39.52643411	19284073	0.0246211		
rs7037813	9	41.43220549	20343227	0.0266384		
rs6475584	9	42.75194764	21801027	0.0151258		
rs10511715	9	44.2991851	23069238	0.0315355	1	
rs10511745	9	46.26151352	24776754	0.027244		
rs10812520	9	49.46357493	27037246	0.0372453	1	1
rs1901565	9	52.22456532	28045900	0.0196802		
rs10757817	9	53.20015556	29182125	0.0184795		
rs867469	9	54.91090352	32373708	0.0356501	1	
rs9407015	9	56.45164088	33835079	0.0162587		
rs4879848	9	57.12844381	34982074	0.0117559		
rs2296777	9	59.69919553	37491844	0.0203853		
rs7853023	9	60.42669762	38762575	0.0136931		
rs10908023	9	64.78908631	66557295	0.0123889		
rs1180109	9	66.83520731	70114821	0.0205098		
rs2147241	9	67.63959961	71832487	0.0229291		
rs2130118	9	68.66944299	73572843	0.0356372	1
rs2094583	9	71.08655219	75492954	0.0222493	
rs17064021	9	75.33427748	77846080	0.0201316	
rs1980918	9	77.57616326	79794756	0.0203144	
rs11788387	9	80.04184296	81700430	0.0199902	
rs2209189	9	81.09559087	83027924	0.0302553	
rs10746763	9	84.00095356	85361111	0.0335212	1
rs4877464	9	89.81610755	88459221	0.0310225	
rs10908907	9	92.37019498	89479138	0.0328053	1
rs7042102	9	95.55028105	91843345	0.0160337	
rs2255840	9	96.69427496	93573328	0.0194212	
rs10759835	9	98.7764824	95626078	0.02162	
rs884886	9	101.252719	98397630	0.0330325	1
rs7039994	9	103.1218998	100130760	0.0230498	
rs320201	9	104.8507613	102020859	0.0232994	
rs7865685	9	106.2208351	103624797	0.0231647	
rs2771036	9	108.2039397	105284817	0.025547		
rs7867544	9	109.6611745	107093535	0.0308434		
rs10739277	9	112.9774042	109131513	0.0432186	1	1
rs7860625	9	118.0883017	113144641	0.0725416	1	1
rs2900611	9	121.7067724	115520094	0.0256866		
rs7047525	9	124.8287657	118150133	0.0231748		
rs16908934	9	126.957769	119587083	0.0227017		
rs12340264	9	128.3894892	121189475	0.0219147		
rs2778635	9	129.0893065	122309353	0.0122725		
rs10125297	9	131.1539299	123839303	0.0190171		
rs871391	9	133.0683706	125499935	0.0147617		
rs516104	9	134.3504142	126659741	0.0225297		
rs7859	9	135.6152456	127783119	0.0237117		
rs2417192	9	139.3090203	129850905	0.0234375		
rs10793870	9	141.8542888	131372492	0.0231974		
rs12376178	9	145.2515431	132430682	0.0159744		
rs3025363	9	147.6888638	133511622	0.0299956		
rs4842145	9	153.1518396	134822894	0.0269781		
rs1009473	9	158.0485383	136003257	0.0360043	1	
rs7090242	10	2.781593849	1628527	0.0349319	1	
rs10508245	10	11.06809723	3143888	0.0261958		
rs1901632	10	15.41553689	4803211	0.0302632		
rs1107345	10	18.34232272	6127301	0.0261639		
rs7921493	10	21.22082735	7379070	0.0432186	1	1
rs11255712	10	23.77272607	8551633	0.0316968	1	
rs1324322	10	27.38858898	10612757	0.0380449	1	1
rs2815611	10	30.59137008	12541404	0.0257618		
rs1409392	10	36.29484723	14661159	0.02088		
rs3012478	10	41.01123841	17075400	0.0212356		
rs993864	10	43.18429509	18579801	0.0214511		
rs10764052	10	44.67401799	19863473	0.0237703		
rs2666771	10	46.18276717	22188236	0.0288998		
rs1414443	10	47.21700082	24155023	0.0216918
rs2367979	10	49.68920218	26020563	0.0246062
rs9416795	10	52.98500103	28749556	0.0218809
rs4747667	10	55.92495755	29876416	0.0292425
rs10508773	10	60.168139	32615450	0.0200364
rs2605506	10	60.70779974	33838752	0.0281936
rs7906685	10	62.60125916	36419509	0.024977
rs12243792	10	63.40058065	38121722	0.0172063
rs2247979	10	64.48889488	43243734	0.0189941
rs1963172	10	65.48608376	44637058	0.0184795
rs4460751	10	67.13186873	47120496	0.0123741
rs4341471	10	67.79717893	48141132	0.0252639
rs10776612	10	68.63148821	49405569	0.0212588
rs751595	10	69.46115838	50634662	0.0210865
rs3011773	10	70.01946612	51946091	0.0170435
rs3862864	10	75.67607597	59582876	0.0209299
rs1684895	10	78.19221963	61396876	0.0243085		
rs4948508	10	80.12581084	63567458	0.0317416	1	
rs3858126	10	81.46901891	65381692	0.0369824	1	1
rs10509245	10	82.50363433	67337890	0.0272473		
rs17457687	10	84.47694597	69581580	0.0191367		
rs4746826	10	85.40806465	70655656	0.0353724	1	
rs7072160	10	92.61335522	73139448	0.0379235	1	1
rs4691	10	93.98531454	75231922	0.0524792	1	1
rs499437	10	96.90668736	78663949	0.0360608	1	1
rs731491	10	99.7209908	80919339	0.0297943		
rs7915399	10	103.3024671	83986972	0.0165356		
rs1857459	10	105.3843866	85674575	0.0367693	1	1
rs12220079	10	107.2328121	88124237	0.0245914		
rs7097946	10	108.9114965	90635321	0.0358949	1	
rs10785942	10	110.1667773	91892878	0.0242462		
rs11186543	10	111.5776304	93109747	0.0341094	1	
rs701873	10	113.3433118	95180558	0.0333327	1	
rs1023331	10	116.0126763	97756527	0.0460697	1	1
rs1060445	10	119.2934749	100208756	0.0248772		
rs2475435	10	121.3331073	102914353	0.0267462		
rs7071247	10	122.4634522	105247776	0.0310459	1	
rs7075669	10	123.926243	107583390	0.0215314		
rs10509826	10	124.8999009	108794830	0.0361676	1	1
rs1936488	10	126.096558	109899733	0.0202131		
rs17127304	10	127.6755631	112124490	0.018044		
rs1325172	10	129.298745	114348545	0.0356825	1	
rs540609	10	132.5151775	116029991	0.032352	1	
rs17094083	10	135.8420235	117850841	0.0253716		
rs927824	10	141.175005	120570843	0.0181561		
rs7913820	10	145.2684756	123085188	0.0278		
rs7906403	10	148.7663544	125099468	0.0284448		
rs7088656	10	151.9905871	126314067	0.0305572		
rs12243508	10	156.6773074	127987718	0.0215587		
rs10829838	10	159.2474404	129048843	0.019557		
rs982836	10	163.610458	130366835	0.0163737		
rs2397769	10	173.491685	133010690	0.030397		
rs11146457	10	177.2697585	134332339	0.0379019	1	1
rs4131364	11	0		802188	0.0322654	1	
rs907604	11	0.899479925	1805930	0.0305308		
rs10832869	11	3.619228507	2867197	0.0276728		
rs2570580	11	7.473998339	4894928	0.0247279		
rs1506981	11	11.0415944	6735647	0.0280378		
rs1105984	11	13.39724273	7973306	0.0292829		
rs2872220	11	16.26342381	9572035	0.0183247		
rs1378357	11	19.96005433	11758937	0.0282342		
rs745361	11	22.04078488	12902779	0.0301822		
rs16930498	11	23.09444682	14713671	0.0216643		
rs2045272	11	27.71270264	18234674	0.0364605	1	1
rs871401	11	34.4605124	20238944	0.0268789		
rs7931276	11	36.53026108	21606387	0.0406167	1	1
rs11027552	11	39.6790394	23816567	0.0212198		
rs1477569	11	41.94057985	25187356	0.0323708	1	
rs4923373	11	43.55627525	26635787	0.018239		
rs7949057	11	44.72633225	28211629	0.0151126		
rs533448	11	46.03235819	29969034	0.021351		
rs224619	11	47.44145167	32021832	0.0195607		
rs3817442	11	48.96928656	33753984	0.0229847		
rs4593985	11	51.99383347	35359700	0.0252073		
rs1030774	11	56.19381661	38454177	0.018937		
rs4755538	11	56.97192426	40004330	0.0169346		
rs10742587	11	57.88573839	41446731	0.024937		
rs10501292	11	58.80001378	42952963	0.016275		
rs4755844	11	61.08986352	44514950	0.0358499	1	
rs2863720	11	63.52638917	46192202	0.0148074		
rs2290149	11	63.68502404	47302492	0.0140664		
rs1603756	11	63.89666497	51171349	0.0173055		
rs9666086	11	63.96303988	55460226	0.0199411		
rs2218868	11	64.20603836	57682553	0.0397084	1	1
rs2237997	11	65.9219696	60528666	0.0417989	1	1
rs12804561	11	68.68101383	62673153	0.0423268	1	1
rs2904979	11	70.66211106	64757295	0.0241507		
rs2282504	11	73.58931283	68457694	0.031301	1	
rs2015997	11	77.38729251	70341866	0.0273871		
rs523907	11	79.44270694	72591286	0.0205019		
rs11824535	11	80.73516926	73832396	0.0232997		
rs629137	11	81.53004373	75536358	0.0291678		
rs728130	11	83.60946611	77182644	0.013152		
rs2508241	11	85.04045088	78559820	0.020552		
rs7342241	11	87.79514752	80385259	0.0317094	1	
rs12806136	11	88.47424889	81392792	0.0185346		
rs11607377	11	89.210561	82495874	0.0131924	
rs11823687	11	90.13663523	84254947	0.0270755	
rs2015747	11	90.74413873	86180785	0.0313924	1
rs4753486	11	92.48022073	87898337	0.0220316	
rs1370018	11	93.59752306	90655656	0.0145591	
rs956267	11	94.77868816	92381834	0.0239742	
rs526108	11	96.70620329	94793821	0.0275473	
rs10791598	11	100.5596245	96588541	0.0206584	
rs2406073	11	101.252358	97884900	0.0173929	
rs2897642	11	102.5756352	99317945	0.0194135	
rs7121108	11	103.9754795	100957541	0.0212258	
rs488753	11	105.2563483	103481503	0.0349856	1
rs10502069	11	106.7693724	105821821	0.028502	
rs1800889	11	107.817287	107668697	0.0215368	
rs317519	11	109.8663789	110395604	0.0287676	
rs1447460	11	112.5722552	112241807	0.0219915	
rs2509220	11	113.8771076	113437670	0.0238029		
rs7131355	11	116.7306457	115582580	0.0345705	1	
rs3802871	11	120.1750354	117281858	0.0176737		
rs643788	11	121.074702	118472968	0.0267583		
rs7940779	11	123.2686176	119924428	0.0220095		
rs11605969	11	124.9614057	120936082	0.0284438		
rs1148085	11	130.0896422	123032279	0.0215793		
rs4936969	11	131.4925816	124395606	0.0394395	1	1
rs649142	11	132.9684939	125548674	0.0278536		
rs7949452	11	135.9876987	126800169	0.028051		
rs4372467	11	136.8758983	127888014	0.0202941		
rs373665	11	145.0190494	130911917	0.0245782		
rs1901916	11	150.0403694	132469509	0.0284871		
rs12221828	11	152.699519	133889790	0.0219132		
rs735295	12	0		417944	0.0296697		
rs1860394	12	7.735028765	3309312	0.0272811		
rs534665	12	14.97835772	5191733	0.0309739		
rs740851	12	17.8088808	6508611	0.0503277	1	1
rs7976721	12	21.93658506	7824902	0.0395256	1	1
rs253155	12	24.25630123	9902102	0.0256076		
rs2961557	12	27.52829504	12000772	0.0270395		
rs1158541	12	31.26685213	13737940	0.0248347		
rs4477487	12	35.48484409	17783827	0.0277374		
rs10770437	12	36.43767689	19190657	0.0320355	1	
rs3983516	12	41.74793693	22017581	0.0139897		
rs1479446	12	43.42708492	23693243	0.0303457		
rs699039	12	46.05673795	25055598	0.038653	1	1
rs4265668	12	49.37052788	27229452	0.0304605		
rs4931161	12	51.81900636	29230133	0.0284236		
rs11613738	12	53.78402016	30777702	0.0226414		
rs12370505	12	55.4723665	32981222	0.0344997	1	
rs4595630	12	55.72764027	34254964	0.0118826		
rs1497050	12	56.40699915	38593865	0.0230873	
rs11180882	12	57.2029592	40071264	0.019279	
rs11181702	12	58.01608912	41500215	0.01819	
rs2408141	12	59.48274035	43789830	0.0144773	
rs10506265	12	60.19010366	44802761	0.0146784	
rs10783299	12	63.1420542	47676944	0.0247128	
rs7299647	12	66.27176489	50830366	0.0191091	
rs4374022	12	70.47844719	53660047	0.0290408	
rs844469	12	72.38663157	55606077	0.0202943	
rs10877071	12	72.92219681	56785193	0.0220116	
rs1391129	12	73.89164153	58278278	0.0214867	
rs795342	12	74.26185542	59751235	0.0218432	
rs10877896	12	75.85180811	61312118	0.0154608	
rs10506538	12	78.01473971	63220192	0.0206853	
rs2088170	12	80.42610589	65068679	0.0325771	1
rs10506555	12	82.01926193	66195617	0.0245664	
rs2860493	12	83.73220897	67209117	0.0385504	1	1
rs10444584	12	85.83824257	69825913	0.0285116		
rs954147	12	86.54217597	71189568	0.0234516		
rs11180619	12	88.20571575	74335105	0.0280953		
rs437413	12	90.07973131	75910709	0.0221715		
rs4842309	12	93.06712845	77906183	0.0242721		
rs7304562	12	94.04948043	78947079	0.0230536		
rs17709807	12	96.03969686	81078401	0.0199559		
rs10161479	12	97.71666801	83858692	0.0192532		
rs10506937	12	98.15347857	85803961	0.0188038		
rs12821256	12	99.34386914	87830803	0.0236323		
rs2723891	12	100.0190054	89460726	0.0388949	1	1
rs11106213	12	101.175161	90510661	0.0144141		
rs11107018	12	103.5875196	92316370	0.0381239	1	1
rs7307510	12	107.534804	94740038	0.0321193	1	
rs12231308	12	108.3407074	96019642	0.037583	1	1
rs2373201	12	111.6914978	98835283	0.0279868		
rs7300149	12	113.0818862	100447047	0.0264626		
rs1178402	12	114.4466174	101554733	0.0148944		
rs4129598	12	116.5450723	103123105	0.0202698		
rs7312155	12	121.3434701	105712920	0.0401696	1	1
rs2228315	12	123.2341061	107520364	0.0291055		
rs991817	12	125.6605379	109873257	0.0384261	1	1
rs11066320	12	127.089272	111369135	0.0325683	1	
rs724026	12	129.0646255	112555413	0.0188684		
rs1955105	12	131.2008126	113807502	0.037379	1	1
rs2650170	12	135.521759	116484475	0.0356448	1	
rs1405050	12	138.2640014	117906433	0.0593941	1	1
rs3815210	12	140.5469242	119042389	0.0497696	1	1
rs2466145	12	141.3606765	120613103	0.0241573		
rs427244	12	142.5697068	123130167	0.0252245		
rs3751178	12	145.1353309	124189237	0.0229064		
rs10744271	12	149.5350797	125589297	0.0254452		
rs11059335	12	153.7230132	126754689	0.0328248	1	
rs2398513	12	166.7041431	129501456	0.0372888	1	1
rs10870468	12	170.9340344	131629032	0.026622		
rs11147509	13	0		19372771	0.0432262	1	1
rs377318	13	6.602664346	21784101	0.0393443	1	1
rs2863937	13	12.75973855	24247710	0.0268616		
rs7983897	13	19.73364779	27339031	0.0294027		
rs912396	13	24.00160223	29341244	0.0248385		
rs9805284	13	28.42040608	30722650	0.0256057		
rs878682	13	31.60358834	32735594	0.0117279		
rs12583395	13	33.23937425	34117778	0.0227801		
rs7322307	13	35.86994873	36248679	0.0263273		
rs400288	13	44.08862241	41759772	0.0243827		
rs7323018	13	47.08203282	43513039	0.035605	1	
rs2985961	13	49.23116636	44969981	0.0269691		
rs17289394	13	50.64262828	46371221	0.0260874	
rs1000603	13	51.95609481	48245269	0.0142539	
rs9526782	13	54.88559568	51059970	0.0222858	
rs9536454	13	55.89059045	53012470	0.028425	
rs10507609	13	56.4275679	55793534	0.0215814	
rs13378962	13	56.62060832	57612750	0.02965	
rs1979558	13	57.10152794	58791375	0.0316621	1
rs7339413	13	58.66126873	61023221	0.0122273	
rs9539642	13	59.11637363	62489052	0.031918	1
rs13379032	13	59.83586317	64358918	0.0316937	1
rs9529163	13	61.55841541	66473567	0.0249854	
rs976211	13	62.41445737	68016099	0.0215843	
rs17086822	13	63.10187777	69823821	0.0165763	
rs4885036	13	67.01268832	72209881	0.0234262	
rs4885167	13	69.92738588	73782166	0.0310527	1
rs2175075	13	72.19321246	75656049	0.0316828	1
rs17069858	13	74.40128213	77909834	0.0197516		
rs8001641	13	75.65592154	79590812	0.0295174		
rs12323203	13	77.05613106	80985782	0.0261371		
rs1999513	13	77.97167541	82965743	0.0291759		
rs9531790	13	78.81238299	84921879	0.029276		
rs9559994	13	79.1313558	85957066	0.0230071		
rs9517465	13	79.46591226	86968034	0.02747		
rs996292	13	80.59001145	88982411	0.0289055		
rs828246	13	81.84570919	90144990	0.021673		
rs10083274	13	83.57784496	91910988	0.0213841		
rs1678386	13	88.20759989	94647177	0.0474068	1	1
rs9556553	13	88.89983666	95754094	0.0345896	1	
rs404437	13	90.31871728	96951800	0.0227202		
rs2297319	13	92.48838246	98159960	0.0273131		
rs13542	13	94.31183091	99436393	0.0205446		
rs4429163	13	98.24500136	101950550	0.0194212		
rs951095	13	103.4887835	104276645	0.0206275		
rs12020838	13	107.7131581	105681508	0.0199302		
rs9520800	13	112.6044901	107581296	0.0240906		
rs4255644	13	118.8079164	109887574	0.0184839		
rs7400267	13	132.1780226	113632689	0.0292906		
rs2039864	14	1.541784857	19565753	0.0138591		
rs4982420	14	10.2042372	20705711	0.0394832	1	1
rs943889	14	15.09691727	22239376	0.0305566		
rs2332505	14	20.52098519	25043242	0.0234848		
rs9783674	14	24.36499241	27516561	0.0260233		
rs11624748	14	25.32835797	29093431	0.0234283		
rs7161558	14	28.18823129	30767915	0.0244228		
rs7152286	14	35.7082105	33346235	0.0340895	1	
rs723204	14	38.70539911	35441383	0.0223609		
rs6571767	14	40.00615266	36470592	0.0230148		
rs1168445	14	41.95324947	37856371	0.0246108		
rs2038281	14	42.57623977	38984948	0.0384449	1	1
rs1475807	14	43.03918969	40093443	0.0153056		
rs11846415	14	43.4476458	41478693	0.0216697		
rs2009767	14	43.94966244	43050453	0.0277022		
rs7142055	14	44.73074367	45056755	0.024673		
rs1022956	14	45.34569514	46631086	0.0217304		
rs3126174	14	46.64681884	49412701	0.0163221		
rs4898706	14	49.28900448	51205106	0.0260175		
rs1954333	14	50.80803203	52982584	0.0256926		
rs10498472	14	54.01485784	54424619	0.0392695	1	1
rs2622324	14	55.36127926	55599846	0.0243674		
rs10130183	14	59.16049705	57768091	0.0237968		
rs8660	14	60.12502692	59009480	0.02738		
rs7492698	14	61.79864135	61809845	0.0337033	1	
rs10140978	14	63.428256	63597824	0.0291055		
rs1953417	14	65.06510676	64948662	0.0125425		
rs3825725	14	66.14355019	67105632	0.0225308		
rs2180611	14	67.20472341	68190238	0.0324395	1	
rs886599	14	69.22506115	70244197	0.0220665		
rs4415952	14	71.56299746	72258906	0.0243237		
rs8003055	14	72.8151617	73883515	0.0257408		
rs2543383	14	75.21060832	75696456	0.0227743		
rs12881440	14	77.94635221	77542483	0.0152843		
rs1531631	14	79.82790171	78694245	0.036925	1	1
rs8003492	14	80.90264818	80155105	0.0318635	1	
rs799099	14	81.9163415	81787150	0.0169026		
rs6574774	14	83.11240314	83685868	0.0218357		
rs7145613	14	83.76945878	85051059	0.0178212		
rs1769463	14	85.80870165	86581817	0.0184486		
rs17126387	14	91.18412978	89371067	0.0361553	1	1
rs12436076	14	93.75644443	90558504	0.0188442		
rs2025072	14	94.90764271	91696891	0.0247581		
rs9671457	14	99.33908866	94086589	0.0341894	1	
rs1950276	14	106.4193389	96650790	0.0184832		
rs748988	14	111.8631352	98855994	0.0221478		
rs7153709	14	117.8684868	101053417	0.0147371		
rs4906226	14	120.9559202	102097112	0.0458697	1	1
rs8013773	14	122.8774028	103706337	0.0218593		
rs2010787	14	124.395632	104761137	0.0148151		
rs1024350	14	133.8598391	106212167	0.016058		
rs999842	15	0	20551713	0.0157777		
rs10519445	15	1.667775493	21657901	0.0290308		
rs2739818	15	6.930149156	23102346	0.0221136		
rs1947745	15	14.96266052	25540093	0.0366125	1	1
rs11634609	15	21.53532315	28000704	0.0401941	1	1
rs2611605	15	25.34898862	30228925	0.0379339	1	1
rs1565936	15	29.72939091	31827788	0.022225		
rs11854468	15	32.81873885	33237841	0.0272929		
rs2467338	15	34.99325513	34547540	0.0215368		
rs6496012	15	38.58394842	36938216	0.0211744		
rs1689605	15	41.97387128	38002353	0.0248101		
rs2277533	15	42.94996774	40352880	0.0283213		
rs2002176	15	43.35075118	42243939	0.0148761		
rs1153860	15	44.20793793	43423876	0.0340517	1	
rs4775379	15	45.19906578	44470086	0.0176378		
rs2009404	15	48.10487209	47004015	0.0126754		
rs2078139	15	49.14127353	48447126	0.029783		
rs1906433	15	51.60971599	51666240	0.0380664	1	1
rs8025686	15	53.60392557	54078467	0.0252201		
rs4646568	15	56.02505373	56131582	0.0270308		
rs10519005	15	59.50748603	57498983	0.036718	1	1
rs16942820	15	61.50831894	58695058	0.0178985		
rs881644	15	65.71127703	61450464	0.0261962		
rs936867	15	66.2038901	62982315	0.0150787		
rs4255740	15	68.21976558	64559061	0.0198131	
rs2292745	15	70.82931771	66411344	0.0352061	1
rs1320205	15	74.52865881	68037936	0.0325222	1
rs6495018	15	78.36456875	70884071	0.018315	
rs3784556	15	79.26711821	72119241	0.0285206	
rs11636245	15	80.18919807	73270805	0.0280059	
rs1801591	15	80.32458884	74365817	0.0191391	
rs4886944	15	81.63380206	75930554	0.0213484	
rs3816282	15	82.928582	77137895	0.020985	
rs10519292	15	85.70230686	78864525	0.0304265	
rs1501371	15	87.27849992	80247495	0.0174916	
rs698486	15	88.96217749	81849596	0.0169665	
rs2122859	15	89.22945527	83271296	0.0209489	
rs2448928	15	90.56987051	84920344	0.0300732	
rs387727	15	94.90252407	87441859	0.0323887	1
rs8025028	15	98.87540994	89826448	0.033622	1
rs3922394	15	106.7956843	91998973	0.0310423	1	
rs8040452	15	114.9746464	93998261	0.0301038		
rs1510058	15	127.9286205	97672532	0.0435692	1	1
rs8043261	15	131.1936169	99096589	0.0325771	1	
rs2072986	16	5.37730872	1631107	0.0312016	1	
rs2908654	16	12.08502505	4939393	0.0294927		
rs1891325	16	16.58833704	6372409	0.0550604	1	1
rs896401	16	23.03326157	8394656	0.0316671	1	
rs2197778	16	25.20692776	10130956	0.02328		
rs949429	16	28.44265794	11283530	0.0360457	1	
rs276639	16	31.18047658	13086517	0.025902		
rs30237	16	34.25444931	14282146	0.0423086	1	1
rs4781732	16	36.64289961	16170073	0.0234962		
rs11647711	16	38.07680472	17476026	0.0225938		
rs2650613	16	39.98205246	18768836	0.0159064		
rs13333521	16	41.93141534	19811583	0.0224399		
rs9930730	16	43.17500565	20924771	0.0156891	
rs8047148	16	44.76068843	22282331	0.0339974	1
rs2238517	16	47.80515082	24247434	0.0201467	
rs7205880	16	50.14407207	25758692	0.0343603	1
rs8046736	16	52.78569796	26850740	0.0256695	
rs8045689	16	54.95201743	28895770	0.0231451	
rs9934579	16	56.33786557	30382357	0.0252844	
rs189378	16	57.11634919	34663360	0.0213636	
rs12446160	16	58.24466781	46915869	0.031156	1
rs4785358	16	59.58890647	48397283	0.0258363	
rs1872678	16	61.11543608	49551265	0.0340949	1
rs3112586	16	64.8105531	51241545	0.025141	
rs6499653	16	67.30268094	52435093	0.0277348	
rs1420228	16	70.72890004	54042674	0.0265266	
rs4784754	16	73.06132921	55629464	0.0273165	
rs9937047	16	75.1156831	56703086	0.0328078	1
rs4238802	16	76.78878554	57705443	0.0317746	1
rs9927943	16	79.57033774	59131895	0.0139766	
rs1834072	16	80.98574986	60955676	0.0274666	
rs8060376	16	81.95084508	62356431	0.0195381	
rs11649437	16	83.97688766	64604861	0.0131529	
rs11649335	16	85.1297049	66993655	0.017382	
rs2305688	16	86.61341	69336645	0.0213175	
rs8063013	16	87.4190108	70914400	0.0236888	
rs974563	16	89.33011519	72283153	0.0279979	
rs8054840	16	91.12248592	73408413	0.0182462	
rs7501326	16	91.79298984	74500397	0.0135607	
rs17770042	16	93.30733226	75903312	0.0131178	
rs3751834	16	97.30171968	77573909	0.0358971	1
rs1110789	16	101.7346152	78997802	0.0295344	
rs4889327	16	104.7183692	80094153	0.0168888	
rs25452	16	108.3419468	81176105	0.0217334	
rs867425	16	116.579842	83256233	0.0260201		
rs6539986	16	124.6159132	85526711	0.0420882	1	1
rs4843807	16	127.9065682	86720111	0.0208047		
rs2353033	16	129.8398164	87913062	0.0254708		
rs7224496	17	5.776756312	1651046	0.021793		
rs9907207	17	7.674707532	2743763	0.0198634		
rs12162152	17	11.66516859	4390219	0.0200174		
rs888326	17	14.70892177	5706077	0.0215891		
rs2253820	17	21.38891948	7988894	0.047648	1	1
rs7215135	17	24.45361729	9105273	0.0370793	1	1
rs758112	17	31.39216507	10839178	0.0247197		
rs8067785	17	33.8654614	11871706	0.0199917		
rs765101	17	39.13814516	13594605	0.0253481		
rs8065832	17	47.16442991	17063052	0.0135561		
rs11655786	17	49.99674978	21014745	0.0172542		
rs7220080	17	50.93976741	22613311	0.0325034	1	
rs12601963	17	52.67907132	25270484	0.0202224		
rs8077806	17	57.11041465	28849229	0.0260233		
rs883416	17	62.2344005	30594554	0.0260564		
rs2411189	17	64.34901599	32083996	0.0189022		
rs685098	17	67.44903246	34322461	0.0323725	1	
rs1859251	17	69.50681973	36350144	0.0221091		
rs4792930	17	71.29744347	38059370	0.027539		
rs8081000	17	71.88762699	39070759	0.021978		
rs7216796	17	73.33516784	40747991	0.0184858		
rs2165846	17	73.64253545	42296365	0.0337208	1	
rs11870879	17	75.35607661	45102647	0.0365125	1	1
rs9303599	17	79.22897176	47932943	0.0256125		
rs7215254	17	79.97060692	49240943	0.0147636		
rs6504989	17	82.43706109	51089023	0.0242702		
rs8080016	17	87.78066076	53309402	0.022578		
rs7210832	17	89.03956272	54962996	0.0124521		
rs758467	17	89.92477425	56669039	0.0135367		
rs196948	17	93.19449166	59511039	0.0362049	1	1
rs12939848	17	97.42193397	62801270	0.0355823	1	
rs2072268	17	98.96961541	63814947	0.0217846		
rs8071270	17	104.3346483	66907543	0.0374547	1	1
rs753281	17	108.0286926	68691048	0.0272048		
rs11869791	17	118.0598991	72844541	0.0299491		
rs7502404	17	122.1907266	74132623	0.0242635		
rs1285293	17	129.9268406	75539371	0.0120387		
rs2606246	18	1.459008775	668847	0.0215309		
rs3810068	18	7.537050518	2836499	0.0193093		
rs11081150	18	14.73170016	4776165	0.0171088		
rs736632	18	18.45555955	5963728	0.0379129	1	1
rs400839	18	28.09842868	8650149	0.0439056	1	1
rs206501	18	34.14319346	10416035	0.0228812		
rs1265930	18	38.83056333	11891135	0.0254499		
rs4797778	18	40.03953825	13457275	0.0269528		
rs3861817	18	40.83846477	14637964	0.0137098		
rs7235524	18	43.24181985	17972261	0.012391		
rs12970899	18	44.57816441	19402861	0.0214446		
rs1786153	18	46.50925532	20752168	0.038642	1	1
rs2010028	18	49.39922421	22645011	0.0170162		
rs11564361	18	51.61254371	24158866	0.0374809	1	1
rs16948113	18	53.44615712	26308259	0.0339851	1	
rs12962411	18	54.60038	27645521	0.0182194		
rs8085075	18	57.0885078	31594247	0.0294864		
rs1539850	18	58.5252126	33332857	0.0301507		
rs978807	18	60.08296939	35152457	0.0158457		
rs2082325	18	61.65934865	37672680	0.0171425		
rs669898	18	62.47895168	38754064	0.0231208		
rs10164078	18	63.30080014	40409771	0.018462		
rs4890673	18	66.08094428	42388614	0.0190575		
rs1393515	18	67.49370402	43460797	0.0124287		
rs506426	18	70.29024011	45103331	0.0288697		
rs1954882	18	72.29240562	46544666	0.0189096		
rs7506794	18	74.76928189	48629590	0.0199233		
rs1451949	18	75.86709732	50441081	0.0161315		
rs1517028	18	80.72771702	54961671	0.0285609		
rs8083511	18	86.93607776	58179635	0.0187592		
rs4941246	18	89.29502516	59953284	0.0322752	1	
rs12965472	18	92.06492838	61823068	0.0227017		
rs17079195	18	94.92050337	64288874	0.0391653	1	1
rs4479346	18	99.67113922	67700989	0.0247452		
rs11151863	18	103.6487295	69270859	0.0356347	1	
rs17211623	18	110.6517468	71332531	0.0219928		
rs4799268	18	117.5950847	73997172	0.0322597	1	
rs12970236	18	123.8851521	76108775	0.02983		
rs4536588	19	0	724437	0.0416257	1	1
rs6510672	19	7.238496514	2416737	0.0356691	1	
rs4807493	19	12.42876327	3582036	0.0235107		
rs1552046	19	17.14954048	5073051	0.0495443	1	1
rs2434444	19	23.98161672	7372037	0.0301502		
rs1982074	19	31.13739223	10529673	0.0247692		
rs8100206	19	31.98768582	11625496	0.017408		
rs1042164	19	33.2541139	13125398	0.0309047		
rs715159	19	36.83727172	14900519	0.0352976	1	
rs4808571	19	41.05661887	17087229	0.0222621		
rs2163813	19	46.39439983	19690101	0.0169993		
rs8111998	19	47.74589379	22533515	0.0413657	1	1
rs1978472	19	47.88135758	23816927	0.0138591		
rs2082455	19	50.26195961	34699475	0.036655	1	1
rs17496703	19	53.55711325	36476435	0.038068	1	1
rs284687	19	57.17675166	38926858	0.0207673		
rs7976	19	61.63314893	40670139	0.0500771	1	1
rs7252868	19	66.01703965	45042569	0.0374547	1	1
rs8109818	19	66.7944376	46152461	0.0143537		
rs1988065	19	68.9184079	48066441	0.0225328		
rs4802234	19	71.34612317	49914440	0.0288488		
rs4804045	19	75.41549542	52479427	0.0282379		
rs10409570	19	83.30854926	55637223	0.023898		
rs10500308	19	89.21841258	56800742	0.023991		
rs8103620	19	94.21730115	58389749	0.0321861	1	
rs1059211	19	104.0242487	60251552	0.0253885		
rs8113086	19	109.2282791	61936028	0.0260294		
rs6515824	20	0.122813918	45122	0.0224452		
rs4814697	20	6.841108641	1811025	0.0316256	1	
rs6076623	20	12.35790755	4084671	0.0498624	1	1
rs805764	20	18.09361716	5624783	0.0250554		
rs4813807	20	21.87826895	6802187	0.0221801		
rs6118234	20	27.08197814	8457458	0.0330706	1	
rs1041200	20	32.47726694	10927521	0.0421743	1	1
rs8116153	20	37.28309249	13182124	0.0376673	1	1
rs6044318	20	43.70581283	16707102	0.0291097		
rs1884783	20	46.74555	18673724	0.0385543	1	1
rs6081756	20	48.49698124	19752868	0.0263748		
rs6137241	20	51.07043248	20997362	0.0199184		
rs2567608	20	52.84282803	22965082	0.0260249		
rs6050867	20	54.8177798	25678968	0.023986		
rs2233826	20	55.2694098	29527003	0.0151154		
rs2057262	20	56.19455156	31150793	0.0254245		
rs224371	20	57.15095949	33538245	0.0251884		
rs6126462	20	59.51080683	35958790	0.0414837	1	1
rs6129532	20	61.78336472	38317602	0.0508136	1	1
rs6102912	20	65.27894027	40636349	0.0301855		
rs6032343	20	69.54152371	43637704	0.0463473	1	1
rs914828	20	71.81567461	45050724	0.0241507		
rs2426063	20	74.80339512	46370455	0.0254875		
rs477627	20	76.58842295	47613465	0.0396589	1	1
rs4811158	20	80.08603498	49383424	0.0318479	1	
rs2041317	20	84.92820132	51797014	0.0392719	1	1
rs6023587	20	87.41918926	52821182	0.0264409		
rs6092326	20	91.46104266	54550144	0.0401448	1	1
rs6026288	20	96.20130039	56472717	0.0301572		
rs3916504	20	98.18811319	57551979	0.046859	1	1
rs6071491	20	107.6827092	59117976	0.0339039	1	
rs6010789	20	120.7631874	61031598	0.022671		
rs2427621	20	129.6414462	62330452	0.025448		
rs2205254	21	4.710046961	14647513	0.0233614		
rs8127304	21	7.773301365	15878737	0.0169488		
rs2776068	21	12.21297852	17799662	0.0204576		
rs977712	21	15.7055288	18992561	0.0370599	1	1
rs1735899	21	18.77540997	20734486	0.0425206	1	1
rs2826910	21	21.05679551	21905087	0.0222573	
rs2828417	21	23.199565	23971962	0.0196612	
rs1847698	21	25.81603302	26188634	0.0164373	
rs242339	21	29.62765391	27754283	0.0241946	
rs1236428	21	30.75037552	28895140	0.0169201	
rs2832643	21	31.76544546	30450088	0.0318584	1
rs558059	21	33.36838816	31732770	0.0280777	
rs2834092	21	37.40354205	33421445	0.0226783	
rs1573303	21	42.43275456	36234040	0.0180157	
rs13049125	21	44.74669341	37266873	0.0229854	
rs2836824	21	48.68275608	39302693	0.0324161	1
rs11702531	21	52.3347899	40399077	0.035426	1
rs2839520	21	61.01873615	42742732	0.0302428	
rs2839367	21	78.8905959	46874816	0.0294445	
rs10483096	22	5.646616505	16926334	0.0284191	
rs5993883	22	9.104230329	18312192	0.016191	
rs658793	22	11.58047771	19321149	0.0158096		
rs5754727	22	14.18434057	20381827	0.0133802		
rs469120	22	16.01260379	21607431	0.0268056		
rs5752173	22	22.55411255	24248998	0.0302666		
rs2144183	22	30.14820709	26161476	0.0251891		
rs5762632	22	32.91075498	27171560	0.0296329		
rs2530678	22	33.96419923	28406269	0.0141142		
rs136245	22	34.59999448	29533098	0.021883		
rs739104	22	36.76693436	31312509	0.0228257		
rs2267249	22	38.11474777	32391400	0.0306163		
rs909704	22	40.13512589	34223884	0.0303497		
rs2235338	22	45.72014117	36290380	0.0390073	1	1
rs7364180	22	49.38892538	40543356	0.0179389		
rs916257	22	50.09529418	41955850	0.0257961		
rs2016518	22	59.07985466	44304438	0.0273216		
rs6008953	22	62.75652495	45421591	0.0188047		
rs7288944	22	65.77362267	46644342	0.0272255		
rs848728	22	70.19953045	48061607	0.0319677	1	
rs877292	22	81.38305168	49297632	0.012614		

a. The most informative markers (IN criteria) were determined by selecting the SNPs based on >300K SNP genotypes in two groups of subjects (150 Ashkenazi Jewish and 125 Northern European subjects).  The 10,000 SNPs with the highest IN values were further selected to obtain those markers with a minimum inter-SNP distance >500 Kb.  These European Substructure Informative Markers are shown to provide good distinction between northern and southern European populations (see manuscript text).				
					
b. The cM positions are based on interpolation using the deCODE genetic map.
