# Supplementary material for: Analysis and Application of European Genetic Substructure Using 300 K SNP Information
Source: PLoS Genet. 2008 Jan 18;4(1):e4. doi: 10.1371/journal.pgen.0040004 (PMC2211544; doi:10.1371/journal.pgen.0040004)
Supplement: Supplementary file 2 [file pgen.0040004.st002.rtf]

Table S2. European Substructure Ancestry Informative Markers distinguishing Northern European Populations^a^												
rs number	Chr	cM^b^	bp	IN								
rs2387698	1	18.12236241	10926370	0.0113192			
rs12731666	1	21.60810266	12458918	0.0117923					
rs3010888	1	22.0925561	12721898	0.0127836					
rs2489772	1	27.10190072	14990067	0.0191633					
rs9442235	1	29.20475673	16138663	0.0118614					
rs3026886	1	39.9813581	21326327	0.0165166					
rs10917268	1	41.38663228	22683124	0.0148817					
rs12568609	1	43.86181783	24412729	0.0131277					
rs7521399	1	52.21388971	32091286	0.0153248					
rs11590681	1	54.29676971	34010096	0.0139303			
rs4308943	1	60.16573808	37916831	0.0142662					
rs1886690	1	61.81382211	38954646	0.0130831					
rs11811998	1	63.64152261	40027819	0.0154312					
rs4660438	1	65.25820013	40720045	0.0151718					
rs213759	1	65.78814598	41262091	0.0188637		
rs1544908	1	76.97170732	55014740	0.0175505		
rs17482565	1	80.52229912	57357443	0.0120585		
rs338926	1	82.50842679	58574031	0.0144114		
rs632853	1	85.60977979	61115888	0.0176979		
rs10889272	1	87.92728983	62114570	0.0150063		
rs4915886	1	89.45089373	63389281	0.0129188		
rs11799558	1	91.53188849	64892484	0.0123616		
rs12129709	1	91.68311121	65168844	0.0148297		
rs12065099	1	91.86183488	65608329	0.0165806		
rs6700849	1	94.61633893	67649772	0.0129181		
rs12030971	1	96.83228585	69143284	0.0152469		
rs17325754	1	97.33002576	69917495	0.016632		
rs10735745	1	102.1618464	76752000	0.0220045		
rs11162802	1	104.4966555	79889968	0.0170696		
rs11579554	1	104.6711838	80156170	0.0212314		
rs11163055	1	105.0611617	80750990	0.0183744		
rs12064002	1	105.464456	81057641	0.0133841		
rs10493770	1	110.0122663	85746885	0.0115813		
rs2151842	1	112.1386077	88140220	0.0125107		
rs11165281	1	118.3232375	95141801	0.0141169		
rs6593652	1	118.6323153	95494554	0.0178215		
rs10783028	1	118.9648803	96082231	0.0131453		
rs2809823	1	122.3630156	100715783	0.0153761		
rs11184219	1	125.2051953	104846449	0.0129991		
rs12094260	1	126.6483016	106519356	0.0121702		
rs6582999	1	127.0692653	107209707	0.0141527		
rs865151	1	129.1682287	109207917	0.0121362		
rs697387	1	130.7647549	110386179	0.020577		
rs7526907	1	131.6978937	110823410	0.0117662		
rs2492501	1	133.9267761	112643298	0.0121073		
rs12127377	1	135.46409	114202015	0.0154717		
rs10923142	1	137.3728098	115916703	0.0116923		
rs6676680	1	147.7551479	151475276	0.0150861		
rs6673923	1	150.788567	154015724	0.0265196		
rs12407446	1	152.3632522	155050135	0.0162234		
rs9330294	1	158.094463	158125080	0.0140603		
rs12404676	1	165.1385637	161763904	0.0151718		
rs858550	1	168.9133525	164211423	0.0130268		
rs2284759	1	170.0146107	165072925	0.0190629		
rs17277113	1	174.8735811	168865978	0.011312		
rs4233167	1	178.1165351	173153930	0.0120409		
rs4652192	1	178.3311592	173479793	0.013174		
rs3806300	1	180.5958936	177204027	0.011979		
rs1689803	1	183.1755774	178893486	0.0114687		
rs7521746	1	190.2330819	187794045	0.0179357		
rs10159191	1	190.3116172	188052843	0.0118012		
rs7538393	1	195.1261044	195477523	0.0176017		
rs6427832	1	196.3643616	197197661	0.0144034		
rs2066284	1	197.1466206	197875431	0.0191522		
rs16854813	1	205.7513174	201662794	0.0138516		
rs12021671	1	209.7776956	204046540	0.0128702		
rs17187133	1	210.3276672	204694875	0.0112613		
rs11119145	1	211.7199993	205437824	0.013962		
rs11119274	1	212.83173	206005094	0.0228529		
rs126280	1	213.3055691	206408219	0.017777		
rs4480424	1	214.9699289	208306510	0.0134945		
rs512458	1	215.8275327	208977288	0.0121556		
rs6695712	1	217.5091016	210220624	0.0126971		
rs1795065	1	218.1309121	210793589	0.0112124		
rs11117791	1	223.0393367	213743983	0.0134755		
rs11118045	1	224.0736085	214720804	0.0115637		
rs608082	1	224.8961442	215517323	0.0207671		
rs12743521	1	227.0317229	218489343	0.0114632		
rs1316883	1	228.9968778	221307379	0.0132851		
rs17319408	1	230.5539247	223425647	0.011238		
rs12091966	1	234.7709892	227549946	0.0137894		
rs12083651	1	238.5550663	229497174	0.0121658		
rs12132550	1	239.1563109	229841350	0.0174021		
rs6586395	1	241.1044169	230955554	0.016652		
rs4568821	1	253.4120023	235792388	0.0198432		
rs12028395	1	256.6994741	236947773	0.0241134		
rs17698283	1	257.7130751	237376416	0.0111579		
rs10158939	1	260.4757976	238549384	0.0265851		
rs4658702	1	268.2195837	241616152	0.0121871		
rs10924440	1	270.4949737	242490226	0.0128266		
rs4072374	2	5.34210513	3080340	0.0135477		
rs17019119	2	9.633879173	3983121	0.0110981		
rs10168477	2	14.46760248	6281047	0.0112793		
rs17745881	2	15.09590521	6619558	0.0169095		
rs17455729	2	27.78153228	10878848	0.0111065		
rs16858789	2	31.99679149	12472003	0.0119628		
rs4832680	2	39.51388877	16998621	0.0166493		
rs13034680	2	41.53251281	19356974	0.0130636		
rs824453	2	41.71086219	19613920	0.0113389		
rs16987794	2	44.04007904	20670389	0.0130461		
rs717966	2	45.65252518	22091887	0.0122816		
rs6742202	2	47.66092194	23628346	0.0163163		
rs1545255	2	47.94796062	24651061	0.0119952		
rs2891409	2	47.98183881	24929008	0.0123558		
rs2304572	2	47.98740152	25264930	0.0134934		
rs7590571	2	51.55715581	29536438	0.0133884		
rs7591141	2	66.4688055	42264682	0.0143929		
rs7598637	2	67.2620746	43013978	0.0115587		
rs1036295	2	73.16922697	47094415	0.011092		
rs17495345	2	75.6680096	50179564	0.0214589		
rs6741326	2	76.3664375	51040936	0.0156425		
rs2552353	2	77.58660898	52521358	0.0135466		
rs10197026	2	78.09931586	53135129	0.0177103		
rs6746287	2	79.06028026	55449748	0.0133106		
rs1345941	2	80.68824098	57347229	0.0185452		
rs7586333	2	82.15110075	59095090	0.0137914		
rs17595617	2	82.53027736	59789910	0.0273122		
rs6727258	2	89.32512253	67151679	0.0174824		
rs13020203	2	89.81314583	67490731	0.0125533		
rs4854502	2	91.81453548	68876729	0.0195445		
rs7585767	2	92.8214982	70249995	0.0196046		
rs6705555	2	96.62060442	71974186	0.0162109		
rs150139	2	99.42440608	74827317	0.0187975		
rs17012485	2	100.9314194	76447414	0.011191		
rs12621435	2	101.2094619	76805511	0.0207741		
rs6712681	2	105.4399186	80435836	0.0111714		
rs1435887	2	106.2193435	81539677	0.0157616		
rs1918098	2	107.811408	84276304	0.0163712		
rs7594872	2	109.254171	85586234	0.011776		
rs1256992	2	112.8364247	97090906	0.0129564		
rs11692435	2	112.8983978	97733872	0.0136565		
rs6542847	2	113.7836281	99031989	0.0209265		
rs10496342	2	114.308582	99869162	0.0137258		
rs10496354	2	115.7204037	101706599	0.01783		
rs894392	2	119.5544059	106248178	0.0119858		
rs921656	2	122.0028509	108345523	0.0208664		
rs3132069	2	126.2868693	114592647	0.0199319		
rs6749268	2	128.845035	118335158	0.016477		
rs1437683	2	129.0745492	119015117	0.0149106		
rs17006704	2	129.1604957	119278139	0.0141346		
rs10496557	2	129.2650455	119603665	0.0120505		
rs1554808	2	135.1227229	123373157	0.0144252		
rs1008645	2	137.5861388	125873477	0.0140501		
rs10174187	2	142.4863731	129917155	0.0131498		
rs6727787	2	144.108066	131490317	0.0178736		
rs6747820	2	146.0921269	133531359	0.0129438		
rs7591709	2	150.4599286	138247317	0.0164272		
rs1446394	2	152.0046763	140110088	0.0119628		
rs12691565	2	152.8975314	141366388	0.0113881		
rs300386	2	153.17102	141822860	0.020545		
rs354690	2	154.8999455	143651450	0.0378195		
rs10496954	2	155.6956695	144503318	0.0126883		
rs3928425	2	156.1155413	144968320	0.0138306		
rs12997609	2	157.3399013	146805147	0.0111321		
rs10171287	2	160.084374	150554961	0.0159558		
rs12618502	2	161.3543389	151635289	0.0180564		
rs1369256	2	162.8467905	153819316	0.012076		
rs2166488	2	166.7510028	157985344	0.0122262		
rs733044	2	167.5981921	159413082	0.0110799		
rs2556097	2	168.0769082	160512568	0.0123568		
rs6432594	2	168.1998152	160790842	0.013345		
rs2231895	2	169.8719623	164294334	0.0121652		
rs2192754	2	176.7985763	171244414	0.0116217		
rs12619236	2	182.2990915	176050020	0.0118012		
rs10930718	2	182.9985904	176568260	0.0117554		
rs6729229	2	186.3289789	181519416	0.0116063		
rs17271148	2	190.4999267	190227728	0.0114609		
rs1882396	2	191.193739	191541518	0.0152739		
rs1997458	2	194.4664422	193925776	0.0144922		
rs2719163	2	194.5026797	194262196	0.0121726		
rs10209427	2	194.5326931	194540834	0.0147122		
rs12993717	2	194.6146334	195301548	0.0131346		
rs1553255	2	195.3904723	196182377	0.0121177		
rs295117	2	198.3192926	200972334	0.0136328		
rs10932040	2	200.8812862	204679203	0.0137798		
rs1978368	2	201.7995615	205326843	0.0119738		
rs969517	2	202.6255883	205884517	0.0207235		
rs861079	2	203.3848085	206414627	0.0137701		
rs13395560	2	204.248233	207344316	0.0156109		
rs11695049	2	204.5880849	207676880	0.0120884		
rs17537169	2	205.0180202	208543289	0.0157123		
rs288057	2	206.387221	210218217	0.0130943		
rs10192315	2	206.5108383	210700913	0.01277		
rs1910863	2	208.6662509	213040582	0.014351		
rs11686077	2	216.1105834	218283535	0.0136037		
rs6752254	2	217.2904049	218884249	0.0157353		
rs4459724	2	221.482645	220860827	0.0128791		
rs1424341	2	224.8335866	222509453	0.0215515		
rs13032261	2	229.3298225	227094096	0.0182092		
rs7587228	2	230.6333217	228002984	0.0145005		
rs2048816	2	231.266248	228352267	0.0113658		
rs7584576	2	236.8118943	231333894	0.0139023		
rs10196343	2	238.7411695	232629865	0.0143289		
rs17866606	2	242.4861776	234722575	0.0152448		
rs1405134	2	243.8823164	235283001	0.0146005		
rs1869092	2	248.8346108	237393437	0.0152163		
rs4233629	2	249.6740107	237886118	0.0129759		
rs6431565	2	251.7818437	238618683	0.0157651		
rs6738275	2	258.1418539	240874416	0.0172398		
rs1523902	3	1.964404827	822139	0.0141727		
rs2872338	3	2.382352546	1137041	0.012516		
rs4684146	3	3.062186022	1399745	0.0136539		
rs6799064	3	4.624968268	1894850	0.0160176		
rs10510254	3	9.298612407	3254498	0.014165		
rs17034929	3	11.28782146	3665193	0.0129212		
rs1866997	3	14.73229889	4769099	0.0136401		
rs17600119	3	19.03960131	6403207	0.014803		
rs2029121	3	23.36329953	8470816	0.0187875		
rs17747739	3	26.87574761	9492577	0.013077		
rs1466835	3	30.78179982	12554944	0.0117766		
rs906671	3	30.96547353	12835988	0.0184435		
rs6767356	3	36.40571674	16329471	0.014903		
rs17043135	3	37.67704727	17165593	0.0211578		
rs10433559	3	37.98901099	17740963	0.0143436		
rs1908984	3	42.02830822	21039517	0.0115198		
rs2060530	3	42.60173894	21492579	0.0119628		
rs13068538	3	43.85498031	22894710	0.0114133		
rs6766822	3	46.60758455	24753694	0.0144021		
rs9873664	3	48.54419827	25994466	0.0142967		
rs1488214	3	49.14769116	26754666	0.0127611		
rs1020042	3	52.91803089	29310050	0.0114902		
rs2218724	3	53.94479993	29900522	0.0125083		
rs4368537	3	56.27507002	31256621	0.011663		
rs6599127	3	65.15437852	40932695	0.0115409		
rs904269	3	66.23758828	42051626	0.0115474		
rs11712075	3	67.64005445	43338209	0.0119746		
rs2023952	3	70.86394238	50079311	0.0155844		
rs1465010	3	74.60482575	54821369	0.0120175		
rs9813516	3	81.70374409	60268044	0.0134704		
rs4688639	3	84.22881496	61564753	0.0162051		
rs1491610	3	95.11611895	70637214	0.0248668		
rs7619189	3	100.8834772	73445841	0.0139933		
rs11706497	3	110.0602577	89621056	0.0180215		
rs13322103	3	110.0953592	90211493	0.0199013		
rs13071953	3	110.3487007	95221384	0.0143598		
rs9290227	3	110.3719916	95691745	0.0138516		
rs6796183	3	110.4269134	97047528	0.0210425		
rs4857192	3	110.5922607	97528441	0.0180364		
rs16839940	3	111.487097	99760387	0.01287		
rs13059876	3	111.9020397	100880846	0.0169141		
rs13096852	3	112.0466793	101392332	0.0140772		
rs1672376	3	113.0050025	103118744	0.0122412		
rs10154899	3	114.3375972	105245144	0.0120671		
rs3804639	3	117.8844908	109273525	0.0139204		
rs1154278	3	120.6204076	113969427	0.0124422		
rs2700230	3	120.8457788	114337029	0.0119436		
rs905570	3	121.9359225	115452113	0.0111393		
rs4688104	3	126.250521	119637216	0.0111456		
rs7637171	3	126.7488563	120499271	0.0219573		
rs626364	3	126.8416337	120755573	0.0134524		
rs2292719	3	127.0761075	121980525	0.0129155		
rs12492695	3	127.3709459	122777805	0.011936		
rs6798997	3	128.4568303	123557394	0.0137736		
rs13071295	3	131.1881767	125828673	0.0174479		
rs2139747	3	133.562563	127870573	0.0126451		
rs567384	3	135.2855067	128976562	0.0122089		
rs11916893	3	136.3084438	130195076	0.0141986		
rs2301570	3	136.7141594	130678361	0.0130225		
rs1497305	3	137.5131283	131630086	0.012023		
rs4854647	3	138.4902875	132731139	0.0181386		
rs4682664	3	142.5961535	135402607	0.0216073		
rs9289539	3	144.3879173	138691423	0.0113365		
rs201739	3	144.50935	138991488	0.0169688		
rs4607068	3	145.5242237	140645692	0.0175983		
rs4234474	3	145.8401757	140910138	0.011191		
rs6782694	3	148.2780676	143110558	0.014632		
rs4974389	3	156.7947042	150386621	0.0158841		
rs1010656	3	157.854931	150887024	0.0130092		
rs6772196	3	160.3168106	152630666	0.011295		
rs7652486	3	161.642476	153653388	0.0119436		
rs6765166	3	161.8640719	153980584	0.0115469		
rs1370031	3	163.3608709	156289349	0.0131509		
rs7615026	3	163.6356215	156635207	0.0197387		
rs12634613	3	164.6171261	158466918	0.0142967		
rs924247	3	165.7935071	160963030	0.019249		
rs6793927	3	166.6231419	162793538	0.011379		
rs2218935	3	167.3520099	165368175	0.0141081		
rs4955713	3	168.4813613	168438621	0.0113202		
rs3851384	3	170.5567948	170484670	0.0139463		
rs1454149	3	181.7667051	177716657	0.0122271		
rs2268933	3	184.8654309	180938313	0.0157123		
rs4521229	3	185.4878492	181973618	0.019979		
rs12488465	3	208.7053027	193394505	0.0142858		
rs9820762	3	215.4499672	195658619	0.0126826		
rs7632151	3	215.9482149	195972793	0.0271615		
rs9855729	3	220.5948564	198632892	0.0116096		
rs445275	4	6.26053587	3779967	0.0116351		
rs10016411	4	7.351209736	4680988	0.0180622		
rs1393861	4	8.95836744	5445893	0.0111065		
rs1801212	4	11.99946213	6420591	0.0150071		
rs3857176	4	15.16534816	7157417	0.0119554		
rs10021708	4	17.49989489	7651745	0.0119858		
rs10002315	4	20.76202991	8238876	0.012541		
rs2241466	4	22.43588057	9835372	0.0198208		
rs6855114	4	22.93739879	10338659	0.0189366		
rs6448638	4	49.51680977	29011832	0.0143146		
rs1512155	4	50.47196512	30137808	0.012515		
rs7673403	4	52.92418838	32696510	0.0118896		
rs6855792	4	53.77892882	34561351	0.0120382		
rs3937773	4	53.98260654	35037214	0.0180767		
rs12710777	4	54.33840531	35680490	0.0121823		
rs7685182	4	61.34924159	40733128	0.0149617		
rs7685881	4	62.08599978	41001884	0.016319		
rs17461905	4	65.97676007	47209993	0.0122632		
rs13132569	4	75.33583109	61431922	0.0123809		
rs12646895	4	75.96246792	62444946	0.0119194		
rs4397025	4	76.81768822	63787003	0.0113819		
rs4148298	4	80.54614281	70645461	0.0128979		
rs722113	4	80.85628892	71343263	0.0117466		
rs12642133	4	84.89184028	75900468	0.0124563		
rs4859864	4	86.77468312	78901506	0.0112488		
rs4693421	4	90.00544049	83376575	0.0173715		
rs12643434	4	90.76012517	84301239	0.014074		
rs11940490	4	91.1414863	84686529	0.0118613		
rs4331742	4	95.21951351	88517431	0.0117309		
rs10016081	4	95.54660861	88997243	0.0130584		
rs2005104	4	98.21073508	90568957	0.0160477		
rs3775479	4	98.61271324	91200149	0.015665		
rs12647859	4	98.84057269	91587757	0.0130943		
rs4414922	4	100.1840047	93046437	0.0112088		
rs11735755	4	101.9435509	95985659	0.0250186		
rs3846456	4	102.468866	96717964	0.0117466		
rs6824429	4	102.8581922	97283088	0.0161461		
rs4699356	4	104.2210314	99898461	0.0163971		
rs3805331	4	105.320479	100691110	0.0187656		
rs4698844	4	107.3216017	103575158	0.0128796		
rs1598859	4	107.4476265	103863637	0.0151118		
rs2176313	4	107.9077618	104694776	0.0144922		
rs2866904	4	110.4456087	107701802	0.0227291		
rs7698580	4	110.9187148	108366771	0.0113608		
rs2726675	4	111.2981446	108902547	0.0129991		
rs244050	4	113.9852858	111578496	0.0116271		
rs227906	4	116.2009128	113187766	0.0118953		
rs17048973	4	121.0215823	118303891	0.0116208		
rs11735972	4	123.5672059	122909978	0.0136407		
rs908822	4	124.847376	125263862	0.0122218		
rs10518452	4	125.4552485	126131419	0.0120602		
rs17820124	4	125.8960134	126912382	0.011222		
rs6842443	4	127.8352226	130513193	0.0116351		
rs1979689	4	128.4486826	131465132	0.0166465		
rs2840131	4	130.023046	133269287	0.0165865		
rs10518627	4	130.7760133	134172137	0.0112293		
rs4443343	4	132.5375868	137112741	0.0155294		
rs2048161	4	141.3210695	147210770	0.0136564		
rs907238	4	145.5915131	152980890	0.0123014		
rs12504106	4	147.0474512	154278184	0.0113608		
rs10517677	4	152.5267619	159087785	0.0119984		
rs6536415	4	153.8502163	160679086	0.0134524		
rs7658820	4	155.4392572	162581086	0.0114193		
rs10517796	4	158.2402099	165268194	0.0147822		
rs4241627	4	161.6849113	168099328	0.0165822		
rs1387329	4	163.1052927	169675938	0.0122696		
rs11731647	4	166.0861332	172381728	0.011191		
rs13143572	4	172.2371696	178280724	0.0254597		
rs12501016	4	172.6899269	178644402	0.0123086		
rs13106298	4	174.5674856	179752662	0.0143721		
rs1379411	4	176.2344304	180569926	0.0134418		
rs6831436	4	180.6603762	182734910	0.0137893		
rs12645288	4	182.0509703	183350223	0.0165824		
rs4391081	4	188.6515239	185349467	0.0208679		
rs7660552	4	192.7555215	186443786	0.0118012		
rs9312336	4	193.4980634	186906842	0.0122588		
rs316582	5	5.597129903	2368086	0.0113572		
rs1691018	5	8.937796683	3297550	0.015665		
rs7701328	5	9.920773869	3939760	0.0167539		
rs16876243	5	15.45330285	5825527	0.0113881		
rs2937785	5	16.82453346	6250044	0.011154		
rs924884	5	17.73362674	6593005	0.012313		
rs16880382	5	21.56227861	8381125	0.0160176		
rs6881628	5	23.80978462	9592540	0.0137261		
rs2214020	5	24.96569908	9905860	0.0168667		
rs16901689	5	29.4989858	11560368	0.0124547		
rs7707491	5	35.41152293	16235201	0.0120492		
rs17651643	5	37.57397157	16945438	0.0122202		
rs10063082	5	43.70935826	24264954	0.012076		
rs13182055	5	45.89016648	26444063	0.015355		
rs6859290	5	47.10456294	27720272	0.0123173		
rs250413	5	55.99402801	34018325	0.011312		
rs1363926	5	59.84317485	37928482	0.0154637		
rs4869578	5	61.17961306	38257417	0.0127466		
rs384267	5	65.35875417	41302975	0.0185368		
rs2456220	5	68.13640406	52202489	0.0123241		
rs17242960	5	68.68925364	52755777	0.0114772		
rs10491367	5	70.03534617	54239231	0.013159		
rs11748140	5	72.88460863	56765369	0.0161574		
rs4394088	5	75.50421644	59663312	0.0138744		
rs2303802	5	78.48025332	65494453	0.0119766		
rs1697143	5	79.13897535	66516825	0.0244237		
rs7720751	5	80.05859311	67270813	0.0148481		
rs17735123	5	85.7662517	73488223	0.0163749		
rs16874127	5	91.77286229	76599527	0.0135605		
rs3846677	5	93.63009226	78125189	0.0111956		
rs10942889	5	94.67801074	78820037	0.0113658		
rs10050980	5	95.3940333	79507139	0.0112289		
rs6452431	5	97.72824731	81166273	0.0160249		
rs10077241	5	101.8821572	85451555	0.0133731		
rs10462465	5	102.8663136	87280658	0.0113053		
rs157237	5	104.344737	89425453	0.0119628		
rs7725438	5	105.3800877	91419604	0.0132756		
rs269850	5	108.5576391	95846052	0.012395		
rs727637	5	110.4557333	98241891	0.01277		
rs32441	5	111.4693317	100132867	0.0154716		
rs17463958	5	117.3779319	110687702	0.015657		
rs10045343	5	118.1215256	111274626	0.01812		
rs10515441	5	118.125874	111563400	0.0152847		
rs1482363	5	119.1078336	113170241	0.0119628		
rs251867	5	120.2351505	115096071	0.0144692		
rs153645	5	121.8691887	115892477	0.0155566		
rs10519510	5	123.7788663	116835987	0.0153315		
rs7709399	5	125.7217666	120796742	0.0133106		
rs2560301	5	126.3508432	121457707	0.0129188		
rs10068902	5	135.4677376	133129405	0.0117397		
rs13359372	5	144.2916546	142593131	0.0134155		
rs6874663	5	146.1835927	143449989	0.0129653		
rs1432770	5	147.2857878	145282859	0.0176229		
rs4705295	5	148.0065899	145769299	0.011979		
rs16750	5	163.3897218	159062555	0.0125538		
rs4628026	5	164.8896468	160166334	0.0136482		
rs4130719	5	168.7346925	163799588	0.0163183		
rs17069574	5	174.963763	167368552	0.0131545		
rs1368406	5	183.6242645	170839889	0.0129181		
rs17660328	5	187.3188687	172273428	0.0112033		
rs4868253	5	188.479858	172653442	0.0166532		
rs2644640	5	194.6104581	174788017	0.0161005		
rs10794696	5	201.367351	178174101	0.0129619		
rs12203592	6	0		341321	0.0643451		
rs9502971	6	4.381515092	1516159	0.0119628		
rs1265203	6	11.53811955	4323996	0.0112356		
rs17141252	6	14.75906465	5711452	0.0148025		
rs6940057	6	19.78675704	7783509	0.0132901		
rs855395	6	23.80389583	10157123	0.013255		
rs2327506	6	28.36049415	12137410	0.0116399		
rs1342744	6	35.19612342	16160498	0.0112848		
rs16885	6	35.69673899	16414730	0.0138748		
rs631661	6	37.24683635	16864985	0.0131618		
rs9465438	6	41.32408338	19666883	0.0142967		
rs201328	6	43.20195627	21154069	0.0138953		
rs952579	6	44.39110206	21992419	0.0129653		
rs943068	6	46.70534146	23527622	0.0120444		
rs807704	6	48.01498339	24408825	0.0127005		
rs1543603	6	49.73542714	25521901	0.0117554		
rs603089	6	49.91717152	25985949	0.0180077		
rs13215804	6	50.79510542	28523551	0.0170255		
rs762324	6	51.24717435	30563865	0.0176229		
rs2844651	6	51.27352095	31008643	0.0123575		
rs9366778	6	51.29534973	31377152	0.0186979		
rs3117582	6	51.32080477	31728499	0.013717		
rs592229	6	51.5089457	32038420	0.0271942		
rs3096700	6	51.74510167	32329760	0.0179157		
rs1794275	6	52.11841738	32779226	0.0150122		
rs622917	6	53.00928445	33810847	0.0213749		
rs4713949	6	55.50845898	36369863	0.0120437		
rs2395653	6	55.90702085	36628371	0.0120698		
rs2622892	6	56.62861948	37362968	0.0149715		
rs9366950	6	57.2411532	38324672	0.0115221		
rs10484854	6	57.55651875	38772572	0.0117662		
rs170644	6	58.00456332	39188842	0.0152418		
rs716953	6	58.53544667	39457379	0.0113608		
rs7764278	6	64.29240965	42265795	0.0125735		
rs7748977	6	66.46105913	43621785	0.0119858		
rs2396081	6	66.87680407	43878490	0.0129829		
rs10948327	6	72.34237895	47174513	0.013255		
rs243099	6	73.35007445	48762363	0.0140199		
rs12202888	6	73.41390781	49284637	0.0178019		
rs12193402	6	75.87034705	52393791	0.0181587		
rs4509106	6	78.8036953	55631183	0.0156349		
rs1755833	6	80.15163276	57294253	0.0147756		
rs11757369	6	81.25633507	64970057	0.0140303		
rs9454096	6	82.75962713	67959331	0.012395		
rs2034781	6	83.29873291	68932219	0.0134541		
rs582962	6	83.67562739	69578699	0.0117077		
rs10942957	6	85.99895734	72393738	0.0122019		
rs1343227	6	86.32390286	72674376	0.0133736		
rs12191488	6	87.67836583	74322596	0.0120383		
rs1572228	6	88.15724838	75285933	0.0144252		
rs2184925	6	88.34146289	75695019	0.0130033		
rs13194998	6	88.73137181	76585158	0.0112062		
rs2798950	6	89.44587888	78255240	0.011617		
rs2803183	6	90.51634596	80235582	0.0130451		
rs6454198	6	91.08060412	81794839	0.011777		
rs12198061	6	93.32046131	86607704	0.0126682		
rs9353512	6	93.54979943	88686472	0.0141064		
rs13200910	6	97.3913358	90850197	0.0128408		
rs9353982	6	100.2431486	93858759	0.0182479		
rs11153162	6	102.0877436	97311015	0.0133105		
rs10484867	6	103.076523	98439288	0.0135177		
rs2180046	6	103.7986472	99556802	0.0221984		
rs1361168	6	105.2798559	101976888	0.0180109		
rs2518201	6	106.0859505	102409773	0.01247		
rs1546963	6	111.8261365	108132839	0.0197959		
rs527507	6	113.1997878	109234577	0.0139463		
rs9400660	6	117.6760917	114130809	0.0209342		
rs1748168	6	117.760523	114955404	0.0111275		
rs3798236	6	118.2223247	116416342	0.0167623		
rs7744432	6	119.6410794	119914299	0.0136845		
rs6569343	6	123.1509576	123779378	0.0202728		
rs1387910	6	123.8890545	124203250	0.0112513		
rs4321843	6	127.4624454	127625101	0.0147527		
rs11754258	6	135.340585	135186315	0.0121301		
rs9385824	6	141.3005187	138520935	0.0119858		
rs17068823	6	144.1377682	139891646	0.016406		
rs6926578	6	145.1339084	142115717	0.0163854		
rs4896634	6	146.2942608	143657195	0.0131151		
rs9283119	6	152.0713752	149228035	0.011086		
rs6939639	6	155.5379218	151617190	0.0156675		
rs1282557	6	159.0890349	153119049	0.012694		
rs270274	6	160.672095	153922650	0.0121301		
rs11966566	6	161.5115963	154699130	0.012076		
rs11759031	6	163.7006165	155607368	0.0193983		
rs2803353	6	166.7316449	158244807	0.0166252		
rs12199314	6	171.2148296	161554794	0.0159637		
rs2272786	6	174.2967288	162773155	0.0140433		
rs500906	6	175.1932307	163408885	0.0153618		
rs9459128	6	177.0195199	164908952	0.0127466		
rs544603	6	177.8063925	165472104	0.0114263		
rs3798315	6	183.7071629	167515120	0.0178517		
rs6455468	6	185.4652595	168203501	0.0160617		
rs4708459	6	185.8989536	168579125	0.0119984		
rs11756439	6	187.6259466	169465662	0.0151524		
rs9348266	6	189.3206224	170545641	0.017216		
rs13221445	7	8.053593564	3366806	0.0154621		
rs17567580	7	15.59568169	7828812	0.0121301		
rs6959225	7	16.91036947	8305744	0.0120816		
rs2189864	7	18.42841938	8875960	0.0129759		
rs972124	7	20.37362136	9666984	0.0141658		
rs1859218	7	22.8636131	11339108	0.0178903		
rs6979324	7	23.51320174	11735360	0.0129802		
rs849734	7	24.73190135	12564727	0.0118101		
rs7785682	7	27.27417869	14196340	0.0121871		
rs7782814	7	29.00802891	15272912	0.0129918		
rs516925	7	30.24811352	16366323	0.0171219		
rs10275488	7	31.0541434	17077018	0.0113263		
rs12111723	7	31.52727623	17386787	0.0113608		
rs11543651	7	32.55825668	18363899	0.0116827		
rs985246	7	33.15252997	18922087	0.0211834		
rs7787246	7	37.93580713	21895747	0.0119519		
rs10486461	7	42.68550836	26290327	0.0115457		
rs1420109	7	47.85394944	29362160	0.0189648		
rs2392147	7	53.30750991	32639963	0.0121866		
rs1035218	7	55.0235825	35309170	0.0238752		
rs1833208	7	57.35631934	36559308	0.0155116		
rs10279772	7	59.75719213	38479624	0.0140012		
rs1558160	7	60.84830682	39283970	0.0131025		
rs6957465	7	62.16325962	40701898	0.012783		
rs12533837	7	64.58623893	42265680	0.0157442		
rs2331175	7	66.76121203	44733163	0.0122871		
rs1294875	7	67.13892713	45024683	0.0162682		
rs10249706	7	68.0313567	45534591	0.0167647		
rs6967514	7	70.22671271	47669398	0.0123829		
rs7802273	7	71.44552388	49466051	0.0216175		
rs10273	7	73.1058716	50858513	0.0112679		
rs7807708	7	73.7800359	51321032	0.0112051		
rs10499748	7	76.06132177	54501024	0.013345		
rs845561	7	76.8230172	55026917	0.0135177		
rs1010779	7	80.92686804	67883605	0.01247		
rs150881	7	86.17343059	73034010	0.0188714		
rs2908201	7	88.10165492	75571776	0.0127232		
rs2192883	7	90.22118362	77741307	0.0126425		
rs12669805	7	92.30460061	79315770	0.0133106		
rs1524600	7	92.72558486	79782954	0.0122717		
rs4728490	7	94.25647741	81180242	0.0135268		
rs9692165	7	94.77714958	81488042	0.0131981		
rs13225753	7	95.20020028	81803174	0.0154312		
rs6949364	7	97.05867054	83047808	0.0138098		
rs1507599	7	98.99112082	85866910	0.0135301		
rs2718324	7	99.82974555	87851151	0.0141629		
rs1017104	7	101.0567438	89687833	0.0117745		
rs42985	7	102.6896782	91043192	0.017147		
rs3750111	7	102.877192	91373574	0.012516		
rs1541335	7	104.4830704	93105827	0.0120366		
rs7798936	7	106.150283	95057904	0.0140537		
rs2724031	7	106.3700047	95678919	0.0129181		
rs6978997	7	107.5970879	97044275	0.0202635		
rs6942609	7	110.5229568	100488915	0.0131733		
rs12538916	7	110.7934599	100771522	0.0123429		
rs9641312	7	112.3877034	102786638	0.0111477		
rs10953428	7	113.0713381	103620814	0.0159211		
rs4283980	7	113.4177469	104019762	0.0115689		
rs212434	7	115.3121678	105115160	0.0127466		
rs10486014	7	116.2244931	105824252	0.0142437		
rs11772445	7	118.0756313	107409109	0.015124		
rs2966487	7	120.6728229	111928896	0.011443		
rs1916977	7	121.4999032	113551257	0.0195047		
rs10230087	7	121.6513654	113848358	0.0120122		
rs10235162	7	122.0273439	114432071	0.0141111		
rs11761305	7	123.5464367	116581861	0.0264375		
rs12706465	7	126.3867662	122257857	0.0170314		
rs17624713	7	126.8441432	123060479	0.0144992		
rs10954006	7	127.1030084	123460679	0.0147969		
rs2402800	7	128.2621119	125075370	0.0122217		
rs10282703	7	128.5180035	125501916	0.0135891		
rs3757791	7	129.0800071	126827019	0.0119628		
rs1450878	7	130.7058749	128811724	0.0164272		
rs17166207	7	136.5634457	131307236	0.018962		
rs11767724	7	140.8430841	135416010	0.0142967		
rs833010	7	141.9743874	136312165	0.0113023		
rs10954638	7	146.771271	138048245	0.011949		
rs1859569	7	148.3854146	139001788	0.0124547		
rs1554690	7	153.9096871	145183981	0.0116568		
rs700278	7	154.1972576	145627300	0.0126449		
rs17173637	7	161.8941206	149967097	0.0122096		
rs9640302	7	165.5436602	150999221	0.0125989		
rs887106	7	172.0341933	153077222	0.0124915		
rs3823518	7	175.5356025	153914057	0.01689		
rs11768706	7	179.2714967	154966549	0.0119628		
rs7809872	7	181.9984076	155711415	0.0124915		
rs2109302	7	186.7870669	157014751	0.0166101		
rs1039621	7	197.1947675	158128545	0.0197959		
rs876356	8	0		607885	0.0163779		
rs11136596	8	5.590337738	3126569	0.0115409		
rs1523250	8	7.240754853	3789239	0.0118364		
rs2616982	8	9.378236577	4468097	0.0200571		
rs6992802	8	12.54301126	5534186	0.0142858		
rs10888101	8	26.21657173	14947165	0.0119628		
rs1495085	8	26.28489304	15342886	0.0174853		
rs7006668	8	28.39776386	16870090	0.0144216		
rs11778372	8	29.31116837	17382789	0.0114632		
rs11204020	8	32.50712569	18991418	0.0161028		
rs2583692	8	35.54368366	20709981	0.0134299		
rs7820789	8	37.24514369	21398897	0.011663		
rs7814885	8	38.59610853	22119076	0.0113015		
rs4278155	8	41.08322924	23052724	0.0142477		
rs4596664	8	44.04111868	25530628	0.0113389		
rs4242426	8	44.51566733	25854996	0.0186209		
rs7006436	8	45.89470509	26909127	0.0129747		
rs11786174	8	47.65271858	28094708	0.0127638		
rs352418	8	48.25464292	28501177	0.0159519		
rs10097270	8	49.62593001	29284160	0.0138856		
rs6992907	8	53.22181015	32347468	0.0168371		
rs6982890	8	53.69692791	32734640	0.0132834		
rs12677633	8	54.23447485	33992280	0.0244215		
rs12156286	8	57.70544723	39581928	0.0130461		
rs11780780	8	60.51234927	41742759	0.0114806		
rs2070713	8	60.9412801	42164812	0.0115689		
rs1863471	8	64.72263754	53153482	0.0127466		
rs10504180	8	68.01651667	56163454	0.0132756		
rs8718	8	69.25973354	58033401	0.017461		
rs10504268	8	71.32184018	59989174	0.0113881		
rs13281284	8	80.48946266	70613856	0.0177219		
rs1822917	8	83.27453833	72430567	0.0125011		
rs7834280	8	84.02475958	72909605	0.0200218		
rs17827858	8	85.92103701	73580589	0.0112608		
rs10108974	8	89.08513558	77588928	0.0124185		
rs11995144	8	89.80289365	79958892	0.0119166		
rs10112398	8	98.03681983	93864228	0.0116708		
rs4735339	8	100.4306867	96043549	0.0118899		
rs4532570	8	101.20476	96662113	0.0124915		
rs2651458	8	102.2182587	97694626	0.0146005		
rs3133719	8	105.1440094	101171944	0.0216963		
rs10095717	8	106.8440474	101940358	0.0163854		
rs12677340	8	112.3566784	106058438	0.0122412		
rs9297395	8	113.8200205	108584549	0.0155294		
rs3115769	8	119.0026031	118814988	0.0191875		
rs7015718	8	119.4175674	119627172	0.0195462		
rs6651164	8	123.1222554	123074121	0.0125613		
rs7460771	8	124.0640415	123546297	0.0115155		
rs17279118	8	139.6700061	131030946	0.0112608		
rs4304313	8	142.1505192	133130328	0.0112008		
rs4897646	8	146.5483859	135034253	0.0141963		
rs2270409	8	157.4739662	141174956	0.0111682		
rs4907404	8	161.1570375	142635782	0.0122264		
rs11988550	8	162.6382249	143387428	0.0117381		
rs636918	9	0		264641	0.0146933		
rs3793504	9	4.461916282	2150105	0.0115758		
rs7864459	9	7.370576786	3430355	0.0120005		
rs4741859	9	7.943664906	3831216	0.0206145		
rs913259	9	12.14247209	4866997	0.0118672		
rs6476962	9	12.70011847	5314922	0.0151098		
rs1339287	9	18.15755176	8223804	0.0131669		
rs2475357	9	22.53677678	10260339	0.0112216		
rs791656	9	25.96267039	12481250	0.0119766		
rs7038373	9	27.06212161	13355907	0.0114997		
rs9407554	9	27.82087528	13841999	0.0118483		
rs7872577	9	33.88077346	16191392	0.0166705		
rs10733333	9	35.4090684	16893446	0.0112976		
rs10733358	9	38.85942746	18943905	0.011372		
rs7855536	9	40.84512419	20001576	0.0118875		
rs7019271	9	41.64255789	20638805	0.0156422		
rs10964808	9	41.91033557	21023557	0.0134721		
rs7852159	9	44.73290177	23565805	0.0124142		
rs7871735	9	45.28246325	24195004	0.0130331		
rs7018906	9	45.73896785	24585311	0.0114389		
rs10511771	9	48.73381968	26288134	0.0165806		
rs1334811	9	49.60327829	27180655	0.0176492		
rs10968042	9	51.57193953	27662182	0.0119488		
rs10969030	9	53.22321892	29241379	0.0125501		
rs1928091	9	54.53891844	31801669	0.0148409		
rs605383	9	54.98191076	32482903	0.0148797		
rs2800257	9	58.34383268	36795874	0.0122832		
rs10868262	9	66.73621212	69759869	0.0145285		
rs10869031	9	67.27229542	71359840	0.0135888		
rs278729	9	68.61012768	73391521	0.0153248		
rs3934253	9	71.36020947	75646367	0.0211136		
rs11138141	9	76.9581685	79145633	0.0151596		
rs12345503	9	77.46325599	79717987	0.01316		
rs10780514	9	79.21214312	81257540	0.0120994		
rs12377016	9	79.96424532	81602982	0.0119194		
rs10780596	9	81.17281718	83190394	0.0148747		
rs11793897	9	81.69920189	83562397	0.0133106		
rs2251530	9	82.33148319	84042494	0.0182266		
rs17088268	9	83.52989197	84989047	0.0116175		
rs2841455	9	83.85826544	85248410	0.0117923		
rs3211663	9	91.27182326	89155642	0.0226959		
rs12554199	9	92.36450782	89477463	0.0124915		
rs7870926	9	100.5880823	97876500	0.0136791		
rs10521057	9	104.2856727	101404100	0.0130019		
rs16936082	9	104.527626	101668177	0.0119628		
rs4743564	9	105.2228786	102427002	0.0156111		
rs10118378	9	109.1866368	106502757	0.0157489		
rs10979066	9	110.9648917	107677074	0.01222		
rs4259484	9	114.8012731	110565557	0.0151287		
rs13295839	9	116.2742353	111718962	0.0113391		
rs7858808	9	117.4591961	112791623	0.0115457		
rs1805313	9	118.5927558	113230745	0.0159773		
rs10982246	9	121.0063226	114329947	0.0185045		
rs16932027	9	121.4878253	114833660	0.0131383		
rs6478230	9	122.7179037	115951295	0.0145934		
rs9299261	9	126.364985	119302461	0.0124577		
rs2184221	9	127.0805591	119646040	0.0126082		
rs945249	9	127.6549152	119921814	0.0116217		
rs16928653	9	133.3701126	125924643	0.0151637		
rs1874777	9	134.3745303	126680556	0.022068		
rs1124602	9	135.2657775	127422546	0.0197152		
rs913984	9	135.5479934	127712696	0.0122216		
rs11791419	9	138.8653335	129557646	0.0114833		
rs4740358	9	140.4712494	130597313	0.0141573		
rs2987380	9	142.4974053	131732952	0.0115471		
rs1964196	9	146.1835961	132884974	0.0138753		
rs2387673	10	2.521340051	1449379	0.0111997		
rs10795076	10	12.83965337	3815343	0.0142437		
rs9423942	10	19.61520059	6689138	0.0116282		
rs1931902	10	21.80672379	7695572	0.0136632		
rs1243974	10	22.77586537	8218558	0.015963		
rs827628	10	25.26080057	9060240	0.0146385		
rs4623797	10	29.48704143	11887247	0.0111065		
rs10795942	10	30.21007648	12315542	0.0148913		
rs11258614	10	34.01147053	13871879	0.0176337		
rs7909464	10	55.27510433	29582635	0.0152555		
rs793096	10	59.69456278	31425455	0.0130525		
rs2284944	10	60.44871854	33530847	0.0119875		
rs1200826	10	63.28957777	37604993	0.0295131		
rs2503848	10	64.30130913	42992791	0.0157403		
rs11101312	10	68.54223615	49277756	0.0117424		
rs10824914	10	69.66334993	51465401	0.0128007		
rs7075768	10	72.29627382	54340014	0.0113608		
rs10825066	10	72.98203672	55004011	0.0122786		
rs1930146	10	73.47782785	55956562	0.013641		
rs16907003	10	74.17680649	56557144	0.0127918		
rs4948235	10	77.83609041	61055737	0.0128418		
rs10761660	10	80.63811727	64186312	0.0157613		
rs1007042	10	85.0080806	70286979	0.0160374		
rs4746826	10	85.40806465	70655656	0.0125819		
rs17497293	10	87.11399014	71241004	0.0149649		
rs1413710	10	89.04051954	72016477	0.0144507		
rs11000000	10	92.73539614	73216067	0.0149086		
rs2616612	10	96.89846051	78657189	0.0135628		
rs11593576	10	99.44406702	80685902	0.0116282		
rs11189831	10	102.2403346	83174129	0.0154048		
rs489466	10	103.7313728	84638946	0.0129907		
rs2616667	10	106.7762944	87323287	0.0118032		
rs12412656	10	108.0298204	89771870	0.0136373		
rs2154254	10	109.1052096	90832411	0.0185851		
rs12249377	10	110.8766528	92582936	0.0135514		
rs1878424	10	111.7790715	93814060	0.0130992		
rs2761291	10	113.2024463	95078170	0.0133499		
rs7094594	10	114.5149273	95726080	0.014062		
rs11812285	10	115.3206669	96750802	0.0233661		
rs725926	10	115.5056147	97236674	0.0183538		
rs10883479	10	120.543877	102152850	0.0116073		
rs717751	10	124.8978205	108793301	0.0115471		
rs2773207	10	125.9561166	109591882	0.0124915		
rs4561129	10	128.4920553	113106988	0.0181785		
rs10885355	10	129.2365042	114248441	0.016156		
rs4918844	10	131.0443107	115309701	0.0119252		
rs639813	10	132.4262627	115998240	0.0157716		
rs2475229	10	133.6748509	116248374	0.0115864		
rs11196967	10	134.6779216	116640629	0.0111234		
rs2901100	10	136.0853885	117999796	0.0160203		
rs6585474	10	140.2004039	119933650	0.0119628		
rs10886671	10	143.8024521	122111778	0.0154689		
rs12570141	10	144.2393816	122438883	0.0139614		
rs7906816	10	146.5390763	123402389	0.0154072		
rs10887047	10	147.0225726	123770790	0.0115523		
rs4751890	10	147.5225923	124151781	0.0156099		
rs2148379	10	159.3555864	129076914	0.0175863		
rs1926150	10	159.8741765	129441662	0.0167081		
rs10491030	10	165.488278	130690227	0.012271		
rs12247354	10	167.753	131326551	0.0116973		
rs7904364	10	173.6825326	133063691	0.0131453		
rs9419387	10	175.5094968	133608348	0.0124142		
rs129074	11	3.257195406	2690701	0.0155028		
rs897111	11	14.12603113	8325991	0.0118062		
rs1496292	11	17.19097283	10791724	0.0177649		
rs10831905	11	21.71942978	12733043	0.0124135		
rs12795982	11	22.73373752	14070894	0.0126099		
rs4403789	11	26.86099941	17687639	0.0146524		
rs2729910	11	29.35978272	19379693	0.0127605		
rs10741808	11	33.05866974	19977460	0.0149764		
rs2468638	11	38.59345624	23041477	0.0116917		
rs10500956	11	39.13808192	23435031	0.0114716		
rs12275853	11	42.02511808	25270610	0.0132543		
rs11603501	11	42.64613515	25843061	0.0120157		
rs2793471	11	45.54765438	29344374	0.0110875		
rs10835941	11	47.73004474	32509711	0.0133767		
rs3781578	11	49.53306148	33863402	0.0121073		
rs1267472	11	50.74811421	34290400	0.0129759		
rs11539202	11	51.32081639	34925688	0.0139928		
rs7934426	11	55.8042884	37270065	0.0130922		
rs2114088	11	58.92359997	43137906	0.0116477		
rs7937428	11	59.69128873	43979187	0.0188421		
rs2298608	11	64.3800187	58161043	0.0121401		
rs10896958	11	64.61955715	58793269	0.017263		
rs1812458	11	66.87433357	61165025	0.0178362		
rs10792322	11	67.2973376	61518128	0.0139933		
rs17158154	11	68.92409113	62876062	0.0163712		
rs11602256	11	72.40737587	67900859	0.0119436		
rs7946208	11	74.49347921	68827005	0.0155908		
rs10431150	11	77.71051308	70644511	0.0114691		
rs7112734	11	81.34294234	75428224	0.0122782		
rs1941603	11	82.39693153	76037370	0.0146605		
rs11607746	11	84.94006114	78542253	0.0134541		
rs10501445	11	85.57223297	78876638	0.0154422		
rs283310	11	86.22510629	79261493	0.0184926		
rs666649	11	88.52901728	81460553	0.011392		
rs494791	11	89.21896804	82674634	0.0161827		
rs1432053	11	89.24994123	82937768	0.011804		
rs12363937	11	89.47069726	83254108	0.0157392		
rs11234095	11	89.97652398	83990352	0.0213492		
rs669336	11	90.31741164	85376515	0.0132031		
rs4944000	11	92.13475579	87137527	0.014941		
rs966149	11	92.39804429	87605719	0.0130544		
rs308793	11	92.48729798	87923538	0.0128366		
rs10128640	11	92.99365545	89726601	0.0115813		
rs504341	11	97.43356324	95190489	0.0111321		
rs17134141	11	102.3660746	99033149	0.0133767		
rs531539	11	102.8209284	99651302	0.0145693		
rs10895115	11	103.950647	100857637	0.0155219		
rs17293152	11	104.4318048	102138344	0.0115503		
rs3847580	11	104.7737344	102695338	0.0149884		
rs1791468	11	106.8134726	105913281	0.0111177		
rs7116354	11	107.5817046	107127988	0.0119858		
rs7123425	11	109.6235178	110226397	0.0146812		
rs472465	11	110.119648	110974172	0.0127419		
rs2574824	11	112.6914977	112573849	0.0212176		
rs12280220	11	113.0187721	112974429	0.0149617		
rs2735202	11	113.9708936	113458316	0.011379		
rs2155854	11	120.4541125	117531885	0.0132457		
rs12282752	11	120.869852	118299128	0.0193376		
rs609305	11	121.5776121	118897864	0.0136707		
rs12796185	11	123.3277667	119952040	0.0120763		
rs4936578	11	124.3829815	120504468	0.0117554		
rs12273605	11	131.7295527	124680691	0.0117645		
rs11221731	11	139.7610518	128877970	0.0124936		
rs4937688	11	147.8565684	131640706	0.0280447		
rs1007018	11	149.9807641	132454683	0.0116271		
rs12279152	11	152.6842175	133861485	0.0121658		
rs2887631	12	4.339212019	1804842	0.0142519		
rs3742076	12	6.178555163	2838430	0.011855		
rs3782814	12	7.25332579	3205386	0.0112289		
rs12319392	12	16.37064681	6040747	0.0162365		
rs7963499	12	26.4589657	11808664	0.0114133		
rs11055697	12	31.51271412	14011756	0.0138669		
rs17762729	12	33.86966224	16044799	0.0133106		
rs12229047	12	48.82931939	26437085	0.0167539		
rs899069	12	49.70534892	27594746	0.0120015		
rs10843894	12	54.17303407	31187486	0.0151338		
rs1259866	12	55.11744038	31998233	0.0148202		
rs2389197	12	55.57509761	33450791	0.018649		
rs12314080	12	57.14586124	39957915	0.0124915		
rs7971883	12	60.24118513	44906568	0.0121658		
rs12372169	12	60.47402363	45576954	0.0127466		
rs11183847	12	61.50896369	46002798	0.0142612		
rs11831940	12	62.30206702	46476433	0.0114513		
rs11169282	12	63.84349802	48816238	0.022024		
rs12427378	12	64.20048399	49360466	0.0177895		
rs4471501	12	64.46719274	49711619	0.0112029		
rs1791648	12	66.69521211	51033060	0.0220826		
rs2280699	12	68.07150665	51783965	0.0115198		
rs2120991	12	69.06434577	52556495	0.0185408		
rs7135506	12	70.42981411	53618580	0.014408		
rs11172147	12	72.67273053	55982944	0.0113785		
rs2047171	12	72.97192616	56995916	0.0135687		
rs4257073	12	73.52300667	57957655	0.0141402		
rs11174549	12	75.76674416	61245404	0.012433		
rs808868	12	76.15585251	61566017	0.0149854		
rs10506479	12	80.06307266	64826291	0.0176229		
rs3925053	12	84.15765023	67718887	0.0151427		
rs1975944	12	84.47126345	68094663	0.0219253		
rs632547	12	85.30930389	69162296	0.0125749		
rs11180273	12	87.85846102	73579360	0.0114364		
rs7487814	12	91.41020226	76637245	0.0112088		
rs10777342	12	101.4598945	90759601	0.0124563		
rs12372697	12	104.1970046	92831989	0.0112556		
rs201430	12	110.6033382	97701666	0.0138918		
rs6538980	12	112.6724494	100195434	0.0153692		
rs11111689	12	115.8549853	102531929	0.0114806		
rs7305812	12	119.8268277	105119787	0.0140768		
rs2101869	12	133.6291571	115634389	0.0214036		
rs1093309	12	135.1973999	116387928	0.0129907		
rs4331170	12	138.299905	117947257	0.0114117		
rs2292702	12	142.2134924	122655008	0.0189203		
rs10846585	12	142.5119002	123053058	0.0167539		
rs897646	12	169.9737876	130790890	0.0142179		
rs10357	12	172.436528	132110961	0.0112537		
rs7336525	13	2.475800897	20816844	0.0115285		
rs2761384	13	4.344494459	21368894	0.0120519		
rs9510565	13	8.368400632	22560702	0.0119554		
rs9551221	13	15.57676777	25234480	0.0162096		
rs7321548	13	17.15332645	26205315	0.0118472		
rs2149973	13	25.0651239	29670079	0.0151746		
rs11147671	13	37.7834079	37243195	0.0153983		
rs9315681	13	40.73246708	38881671	0.013429		
rs4943731	13	41.45938091	39610941	0.0110948		
rs7329258	13	45.57515936	42842759	0.012824		
rs7996510	13	49.63458838	45368378	0.0122696		
rs7321658	13	50.38675231	46111173	0.012154		
rs6312	13	50.64028421	46368825	0.0126296		
rs1360646	13	56.36093194	55167399	0.0114548		
rs9537662	13	56.49285453	56734345	0.0209196		
rs4886026	13	56.55208052	57141569	0.0140278		
rs9538094	13	56.74982607	57997784	0.0120659		
rs17057633	13	57.53866144	59577372	0.0199856		
rs7998134	13	58.11140856	60315035	0.0196939		
rs1412471	13	58.92671777	61365103	0.0116646		
rs2669331	13	59.11730354	62497279	0.013226		
rs12585880	13	59.43154736	63535652	0.0115617		
rs11841049	13	59.54402377	63881065	0.0163854		
rs1889085	13	60.35270133	65029337	0.0154853		
rs17583414	13	61.16960916	65996856	0.0176229		
rs11839068	13	66.46833471	72014214	0.0122632		
rs1409590	13	70.30793898	74038383	0.017452		
rs1772579	13	76.20577404	80086174	0.0131025		
rs1333592	13	77.71711213	82096952	0.0132031		
rs9575619	13	78.39843308	84101690	0.0122412		
rs4129526	13	81.66517715	89865302	0.01524		
rs12018676	13	81.93262252	90289722	0.012068		
rs12867481	13	83.80978576	92096524	0.0137506		
rs1951797	13	85.97109404	93854769	0.0125509		
rs9556820	13	90.81353764	97230391	0.0168201		
rs7320534	13	96.281014	100648474	0.0145296		
rs9300717	13	97.77043133	101634002	0.0124547		
rs9514044	13	98.47181211	102143466	0.0125189		
rs2765584	13	98.79353341	102625828	0.0122397		
rs9300943	13	102.6149951	103985970	0.0167234		
rs4771561	13	109.5448852	106682364	0.0170429		
rs1925391	13	112.5181203	107555920	0.0172053		
rs12874003	13	118.338402	109749316	0.0143681		
rs1928027	13	122.1137293	110897566	0.0163212		
rs9562077	13	135.7938087	113936115	0.0122089		
rs2295682	14	15.2659563	22444702	0.0126845		
rs17527048	14	20.38276566	24951684	0.0121658		
rs4981674	14	24.5145226	28140136	0.0113214		
rs10148024	14	24.94496662	28729886	0.0174989		
rs1950221	14	31.91148319	32260976	0.0130831		
rs8005808	14	36.48937988	33661046	0.0123285		
rs2022725	14	40.13894925	36561587	0.0120884		
rs1168553	14	42.00378692	37934281	0.0196763		
rs10498377	14	43.74418404	42482338	0.0124027		
rs1815619	14	48.79084874	50867994	0.0130083		
rs2145449	14	49.21345561	51154060	0.0139695		
rs7140955	14	50.79970911	52943242	0.0124326		
rs241529	14	55.32525888	55581216	0.0132588		
rs10143058	14	58.32069117	56904596	0.0134137		
rs4902059	14	61.14888052	60971079	0.0142841		
rs8021670	14	61.41441961	61318278	0.0128504		
rs12589358	14	61.97258385	61988607	0.0190782		
rs9788522	14	62.79104681	62752331	0.0162365		
rs4902562	14	66.4948405	67801211	0.0114127		
rs11622517	14	75.44540508	75835500	0.0134236		
rs17751406	14	77.60309018	77005693	0.0217298		
rs8015618	14	77.88117314	77450143	0.0164224		
rs2110420	14	78.63570482	77991106	0.0143062		
rs2113574	14	79.08089779	78253778	0.0145062		
rs4903820	14	79.67448446	78604005	0.01689		
rs6574859	14	84.05565814	85428320	0.0131046		
rs7153598	14	88.66677616	88739707	0.0127918		
rs7152796	14	89.90735719	88994273	0.0145925		
rs3742673	14	92.24532805	89805460	0.0142338		
rs7154324	14	97.27930717	93165928	0.011581		
rs1187763	14	102.2661709	94957664	0.013028		
rs8021112	14	106.5304216	96699773	0.0121259		
rs17508376	14	108.8807632	98028056	0.0144992		
rs7143468	14	121.5804505	102307531	0.0153504		
rs11851852	14	123.6447735	104239473	0.0167382		
rs10047997	15	5.615690824	22657843	0.0152455		
rs4780267	15	21.49928058	27975237	0.0128173		
rs11635677	15	22.64550315	29191625	0.0176129		
rs6494039	15	25.009816	29979194	0.0111556		
rs8034124	15	26.32168524	30936351	0.0154181		
rs613479	15	30.46127372	32146482	0.0121835		
rs12906896	15	32.10088905	32893860	0.0157353		
rs4586385	15	36.83583424	35633096	0.0124577		
rs1604528	15	37.46785125	36005449	0.017526		
rs4334271	15	42.62410589	38423749	0.0135177		
rs2279578	15	42.66394429	38951636	0.0206625		
rs1197701	15	42.8698356	39974784	0.0119277		
rs7171366	15	43.97116844	43173948	0.0149618		
rs12904370	15	48.99824106	48202177	0.0112348		
rs497321	15	51.01236623	51191215	0.0132235		
rs11629910	15	51.39184051	51473913	0.0219914		
rs12441839	15	64.06328704	60359314	0.0127765		
rs1477798	15	67.29666114	64239300	0.012924		
rs8027997	15	68.27046129	64654601	0.0196252		
rs11632449	15	72.11509128	67006398	0.0113752		
rs11635749	15	74.61568757	68073971	0.0127611		
rs4776567	15	77.27933938	69756906	0.0131779		
rs16957064	15	78.10874509	70614105	0.0146622		
rs12439063	15	78.3708486	70937219	0.0129907		
rs12440104	15	78.37984781	71440062	0.0141542		
rs4887111	15	78.79238642	71815338	0.0129829		
rs8024685	15	83.1478264	77339419	0.012927		
rs7172022	15	85.80091271	78913962	0.0211621		
rs4887443	15	89.42864612	84312988	0.0136645		
rs11634465	15	90.57987683	84925312	0.0119928		
rs11634215	15	91.73169738	85559990	0.0129619		
rs10520656	15	92.24203687	85910263	0.0113584		
rs8037001	15	94.76618008	87366490	0.0140772		
rs99521	15	102.8300116	91029637	0.0111377		
rs6416581	15	104.909288	91537878	0.0131538		
rs2127247	15	111.1002594	93056906	0.0141356		
rs2684796	15	126.1182142	97300915	0.012619		
rs11854173	15	133.3104015	100021401	0.0113608		
rs760560	16	3.339778781	888967	0.0118836		
rs2270834	16	12.29438754	5050272	0.0132002		
rs4786780	16	13.72123805	5809388	0.0114263		
rs1463242	16	15.04128505	6121852	0.0137082		
rs7186783	16	17.10995947	6525647	0.0132851		
rs10163400	16	17.86037848	6783832	0.0160552		
rs12149783	16	19.05999491	7399524	0.013255		
rs17680913	16	24.23870922	9192461	0.0147558		
rs12596290	16	29.79760026	12540409	0.0133106		
rs153785	16	35.87300217	15500793	0.0116631		
rs1377554	16	37.3866979	16848739	0.0135384		
rs4781927	16	38.43233766	17803882	0.014378		
rs11644921	16	45.22485463	22680826	0.0123086		
rs2238508	16	47.72901611	24213671	0.0115198		
rs3024536	16	53.48555666	27260214	0.01222		
rs9939450	16	54.02922967	28208988	0.0205489		
rs6565261	16	54.87313825	28811157	0.013172		
rs4387618	16	56.33922663	30383817	0.0119766		
rs957881	16	58.17958399	46751524	0.0148768		
rs4785173	16	59.02881063	48052382	0.012515		
rs7204868	16	59.72766577	48541854	0.0142437		
rs1874494	16	60.92512074	49479621	0.0145506		
rs9938659	16	64.36691166	51002320	0.0134299		
rs1861532	16	68.58446479	53235014	0.0172918		
rs1486731	16	69.67936575	53744492	0.0126207		
rs3790116	16	71.87337755	54945545	0.0130605		
rs7204900	16	74.19385029	56203008	0.012253		
rs17487804	16	82.90821196	63371745	0.0232281		
rs8058014	16	88.02182588	71415045	0.0155995		
rs16972092	16	89.01988189	72112611	0.0119766		
rs9923061	16	91.99588332	74881862	0.0121508		
rs8048863	16	92.69401697	75170251	0.0124683		
rs11642715	16	99.12107448	77904541	0.0141169		
rs7205649	16	100.6072972	78497005	0.0122393		
rs4889126	16	101.3405916	78852188	0.0176229		
rs8044334	16	103.9361051	79806436	0.0130525		
rs6564940	16	106.0854775	80521305	0.0120306		
rs3936367	16	108.1032477	81106786	0.0184683		
rs692612	16	113.0341387	82377400	0.0190006		
rs11642445	16	118.0443803	83646696	0.01198		
rs12923978	16	120.8693464	84503825	0.0117096		
rs4843426	16	123.7287061	85300922	0.0150677		
rs4843507	16	124.9611377	85614547	0.014549		
rs16943315	16	127.2564833	86427141	0.0115333		
rs4395073	16	134.6440657	88530183	0.0161489		
rs4636972	17	3.151832963	1045474	0.0153414		
rs400688	17	12.34976892	4780674	0.0135335		
rs907942	17	17.36144705	6340611	0.0160475		
rs9912143	17	22.76742019	8647754	0.0116922		
rs10468473	17	34.31725487	12068401	0.0142255		
rs9905820	17	35.5223984	12578096	0.0125511		
rs3935801	17	46.34659847	16284495	0.0128992		
rs7221778	17	48.50388281	18728943	0.0112677		
rs373531	17	53.66916389	27011628	0.0112577		
rs12452093	17	58.04707457	29114217	0.0115756		
rs7502772	17	60.62717357	29723177	0.0115046		
rs3760440	17	64.90530252	32302414	0.0148325		
rs17590228	17	67.38390619	34290556	0.0146812		
rs1009728	17	69.05648261	35862733	0.0111024		
rs8077267	17	72.76704754	40411244	0.0112019		
rs4248914	17	73.24024415	40692263	0.011086		
rs17590785	17	78.93586872	47345110	0.011312		
rs7225060	17	79.79774378	48929613	0.0124827		
rs8066468	17	81.66026276	50813403	0.0114519		
rs10132	17	87.94490766	53523370	0.0133706		
rs2047153	17	93.25407716	59623901	0.0115857		
rs1468329	17	94.24518685	61003783	0.0153091		
rs4968938	17	99.60429521	64242851	0.0141169		
rs17780310	17	106.3293217	68204290	0.011581		
rs753281	17	108.0286926	68691048	0.0137261		
rs569908	17	117.49801	72637227	0.0165028		
rs2871647	17	120.7976197	73649072	0.0143062		
rs691127	17	121.7120894	74008709	0.011762		
rs3934711	17	133.9043189	77143096	0.0117466		
rs566821	18	5.20958503	2094793	0.0152769		
rs16943989	18	7.748999878	2882474	0.013867		
rs6506515	18	24.25672454	7493516	0.0121204		
rs9646474	18	40.00224068	13402155	0.0138575		
rs550010	18	45.68012424	20334380	0.0131174		
rs1348338	18	53.46800259	26364571	0.013159		
rs4427879	18	55.73055293	29658749	0.0120752		
rs4621050	18	55.83484855	30156128	0.0150276		
rs1355829	18	60.40847203	35696875	0.011765		
rs637001	18	65.23180838	41820643	0.0117766		
rs8094458	18	69.3790985	44527491	0.0132692		
rs1944362	18	76.02112036	50721343	0.0115788		
rs12327342	18	78.61605307	53457158	0.0123792		
rs937816	18	80.39374361	54607418	0.0132788		
rs564968	18	86.56413609	57829427	0.0136793		
rs11152343	18	86.99705759	58237240	0.0197659		
rs10083961	18	89.60584672	60317371	0.0137893		
rs470490	18	90.9366121	61182216	0.0117753		
rs10515998	18	91.73531688	61521202	0.0114126		
rs936110	18	92.98271002	63227575	0.0167539		
rs4891825	18	98.03616212	66018643	0.0119766		
rs17264096	18	108.4893889	70958244	0.0165548		
rs1553664	18	112.8484859	72420744	0.0129152		
rs4890891	18	114.7335258	72940917	0.0116497		
rs2135091	18	117.6611538	74022466	0.0138753		
rs945704	19	18.57616873	5655353	0.0142437		
rs2335629	19	19.81739077	6154146	0.0146278		
rs875569	19	31.1379369	10529953	0.0115911		
rs1423089	19	32.35529999	12450726	0.0128764		
rs7254735	19	37.75188514	15124385	0.0158016		
rs3813135	19	38.17346409	15448345	0.011662		
rs4808416	19	38.60849965	15928789	0.0116087		
rs1122821	19	45.53979404	18911957	0.0149076		
rs1230313	19	47.73893527	22467258	0.0122221		
rs7257017	19	51.4279449	35468876	0.0125296		
rs7257183	19	54.39828841	37155418	0.01443		
rs2868146	19	55.69002055	37741887	0.0120331		
rs1688015	19	60.8162468	40303753	0.0124981		
rs11881242	19	62.22377409	40975687	0.0124915		
rs10403583	19	64.29491764	43983611	0.0117745		
rs1131620	19	66.59333495	45809709	0.0140038		
rs204538	19	71.11232933	49647767	0.0114042		
rs7257916	19	71.69972832	50174724	0.015772		
rs8109759	19	74.51955444	51641502	0.012924		
rs10419661	19	74.90800503	51965108	0.015513		
rs3764622	19	80.28655464	54198202	0.0177047		
rs3745509	19	82.41148799	55488717	0.0136383		
rs2691244	19	86.36484051	56277071	0.0118205		
rs12327639	19	92.43192492	57758669	0.0178362		
rs17273267	19	100.2670636	59401140	0.0145376		
rs1325156	19	102.5358148	59908762	0.0123616		
rs2742059	19	104.1177384	60342800	0.0200588		
rs2058131	19	108.6673224	61733570	0.0111683		
rs260445	19	117.5383332	63437743	0.011154		
rs282152	20	2.600673809	527657	0.0128041		
rs743082	20	6.524710762	1638191	0.0114806		
rs4814983	20	7.6375111	2065462	0.0145025		
rs6133219	20	17.11748468	5443069	0.029483		
rs6076954	20	20.34131719	6085310	0.012924		
rs6105221	20	38.03040947	14119905	0.0116175		
rs201930	20	44.73980468	17471989	0.0203962		
rs2876507	20	47.17951914	18941114	0.013159		
rs2328492	20	49.15212128	20040843	0.0134468		
rs6076248	20	54.36322663	24587916	0.0131984		
rs845787	20	54.83399502	26145931	0.0135505		
rs4911442	20	57.11098464	32818707	0.0111464		
rs6066892	20	58.74287898	35635762	0.01558		
rs10485678	20	63.25181342	39096743	0.0129947		
rs1475048	20	63.286472	39712523	0.0113658		
rs6030839	20	66.49117782	41467102	0.0149221		
rs6031215	20	67.65425525	41963296	0.0130605		
rs6073394	20	68.4532032	42345476	0.0115221		
rs11906231	20	74.02378581	46062004	0.015261		
rs16997896	20	83.51704179	51346815	0.0116722		
rs11698898	20	84.98092126	51811441	0.0126524		
rs6092900	20	101.5302318	58217893	0.0143436		
rs6061663	20	109.6185376	59401180	0.0162365		
rs6089457	20	111.4584098	59670346	0.0118157		
rs6089784	20	119.6586393	60870007	0.0111598		
rs6090443	20	124.8140971	61624230	0.0134484		
rs6010652	20	126.6656511	61895105	0.0127136		
rs2822548	21	4.461934051	14577923	0.0112525		
rs2824976	21	15.54642291	18948707	0.0255061		
rs2408239	21	21.30034066	22254497	0.0136855		
rs2827845	21	22.21485577	23386781	0.0116709		
rs7278046	21	27.76769706	27093711	0.0130837		
rs992499	21	28.8192919	27463501	0.0140772		
rs2831507	21	30.31581888	28406326	0.0230049		
rs2832146	21	31.04105531	29246626	0.0184783		
rs915538	21	38.34937318	34077953	0.0115523		
rs9981318	21	44.39383693	37071260	0.0182059		
rs2836604	21	48.03654511	38963165	0.015077		
rs4816612	21	48.97444402	39427896	0.0132445		
rs4818059	21	50.08990218	39864658	0.0170234		
rs16999738	21	53.12441721	40702268	0.0142967		
rs980274	21	54.31952985	41134108	0.013258		
rs2839475	21	59.82239213	42481242	0.0129707		
rs4920104	21	60.99520182	42737588	0.0125362		
rs2839589	21	62.60245985	43147296	0.0126099		
rs12626954	21	64.25014929	43691341	0.0150485		
rs2839349	21	78.37173569	46841625	0.0122345		
rs4819591	22	3.301757017	16321925	0.0111065		
rs385773	22	9.661228118	18539143	0.0134974		
rs5751691	22	17.44784792	22190872	0.0118311		
rs5761163	22	22.95348447	24463932	0.0174856		
rs2331295	22	26.60596896	25420266	0.0241343		
rs483241	22	29.220763	25967856	0.0121684		
rs1012769	22	30.9519806	26300757	0.0116636		
rs9613630	22	32.9317866	27196124	0.0118056		
rs130539	22	36.99088637	31501004	0.0130943		
rs137296	22	37.2922085	31824865	0.0147927		
rs9623117	22	49.14599429	38776619	0.0147927		
rs133074	22	49.20449863	39402973	0.013876		
rs767249	22	51.23198073	42438714	0.0120402				
rs135551	22	61.43843197	44873540	0.0120671				
								
a. This set of markers determined using In differences between subject groups chosen using the first PC in ""northern European"" subjects (see text).								
b. The cM positions are based on interpolation using the deCODE genetic map.
